# Epidemiology of Foot‐and‐Mouth Disease in Goats in Uganda: A Risk‐Based Approach

**DOI:** 10.1155/tbed/2808139

**Published:** 2026-04-15

**Authors:** Benedicto Byamukama, Asfor Amin, Frank Norbert Mwiine, Susan D. Kerfua, Abel Bulamu Ekiri

**Affiliations:** ^1^ Department of Comparative Biomedical Sciences, School of Veterinary Medicine, Faculty of Health and Medical Sciences, University of Surrey, Guildford, GU2 7AL, UK, surrey.ac.uk; ^2^ School of Biosecurity, Biotechnical and Laboratory Sciences, College of Veterinary Medicine Animal Resources and Biosecurity, Makerere University, Kampala, Uganda, mak.ac.ug; ^3^ Department of Virology, Animal and Plant Health Agency, Woodham Lane, New Haw, Addlestone, KT15 3NB, Surrey, UK, vla.gov.uk; ^4^ National Agricultural Research Organisation, National Livestock Resources Research Institute, Entebbe, Uganda, nalirri.go.ug

**Keywords:** biosecurity, FMD, goats, livestock, policy, risk factors, seroprevalence, small ruminants, Uganda, vaccination, wildlife

## Abstract

Foot‐and‐mouth disease (FMD) is a highly contagious viral disease with major economic implications for the livestock sector in Uganda. Although cattle are the primary focus of FMD control in Uganda, small ruminants are also susceptible and interact with cattle in mixed species grazing systems. Little is known about the epidemiology of FMD in small ruminants. The objective of this study was to assess the seroprevalence and risk factors associated with FMD in goats in Uganda. A cross‐sectional study was conducted in five high‐risk districts with a history of FMD outbreaks. Blood samples were collected from nonvaccinated goats across 80 farms, and a questionnaire was administered to collect data on animal‐ and farm‐level characteristics. Serum samples were tested for FMD virus (FMDV) nonstructural protein (NSP) antibodies using ELISA. Descriptive statistics and univariable and multivariable logistic regression analyses were performed to identify factors associated with seropositivity. The overall animal and farm‐level seroprevalence was 19.8% (165/832) and 48.8% (39/80), respectively, and within‐farm prevalence ranged from 0% to 100%. Kiruhura district had the highest seroprevalence (32.4%). Key predictors of seropositivity included proximity to a national park (OR = 14.37, *p* ≤ 0.001), district (Kasese: OR = 0.009, *p* ≤ 0.001; Kiruhura: OR = 0.052, *p* ≤ 0.001), and recent FMD outbreak history (FMD < 6 months ago: OR = 6.63, *p* ≤ 0.001; FMD > 1 year ago: OR = 2.19, *p* = 0.009). The most reported challenges to FMD control included inadequate vaccine supply, communal grazing, uncontrolled animal movement, and weak enforcement of quarantine measures. This study demonstrated substantial exposure of goats to FMD and highlighted the underrecognized role of small ruminants in FMD transmission. Integrating small ruminants into national FMD control programs is a logical step given the evidence. For example, inclusion in risk‐based vaccination, strengthening of vaccine supply systems, and enhancing of farm‐level biosecurity.

## 1. Introduction

Foot‐and‐mouth disease (FMD) is a highly contagious viral infection caused by a nonenveloped, positive‐sense single‐stranded RNA virus belonging to the family Picornaviridae and the genus *Aphthovirus*. The causative agent, FMD virus (FMDV), is genetically and antigenically diverse, with seven distinct serotypes including serotype O, A, and C [1, 2] and serotypes SAT 1, SAT 2, SAT 3, and Asia 1 [3, 4]. FMDV affects a wide range of hosts, with more than 70 species of cloven‐hoofed animals being susceptible, including cattle, pigs, sheep, goats, and African buffaloes [5–7]. The infection is primarily transmitted through inhalation of droplets, ingestion of contaminated material, or contact with fomites [8, 9].

FMD infections can present either clinically or subclinically, with low mortality but high morbidity. Clinical severity varies among species, influenced by the viral strain and host susceptibility [10]. In cattle, FMD is characterized by vesicular lesions, fever, lameness, and reduced productivity [11], with calf mortality rates of 2%–5% [12, 13]. Deaths primarily result from acute myocarditis, particularly in juvenile ruminants, whereas adult fatalities are rare. The vesicular lesions caused by FMD can severely limit the ability of infected cattle to graze and drink, leading to weight loss and decreased overall health [5], and can reduce milk productivity by 80% [14]. Beyond direct production losses, FMD results in substantial economic costs due to disease management and control efforts, including expenses for vaccination, quarantine measures, and trade restrictions [15]. Knight‐Jones and Rushton [15] estimated the annual global economic impact of FMD to be between US$6.5 billion and US$21 billion, highlighting its substantial effect on livestock industries. In small ruminants, FMD is often subclinical but can manifest as lameness, fever, depression, neonatal mortality, and vesicles around the mouth and feet [11]. FMD‐related mortality in small ruminants can reach 4.9%, as reported in Iraq [16].

The epidemiology of FMD is complex due to its highly contagious nature and wide host range, with the virus capable of establishing a carrier status in different host species. Cattle can remain persistently infected for 2 years [17] or up to 32 months [18], small ruminants for 5–9 months [19, 20], and African buffaloes for up to 5 years [21–24]. Although persistently infected animals or carriers have demonstrated limited capacity to transmit the virus under experimental conditions and natural settings [18, 25, 26], transmission is more likely when the ratio of carriers to susceptible animals increases [27]. The transmission from career species (African buffalo) to cattle under natural and experimental conditions has been demonstrated [28, 29], although to a limited extent.

In Uganda, FMD is endemic, with frequent outbreaks occurring annually [30–41]. FMD control efforts in Uganda are complicated by husbandry practices such as mixed grazing of cattle and small ruminants and wildlife–livestock interactions in specific areas. Emergency vaccination is the primary response to FMD outbreaks; however, current efforts cover only 1.1% of the estimated 44 million livestock eligible for vaccination [42]. The vaccination campaigns focus almost exclusively on cattle, while other susceptible species, including small ruminants and pigs, remain largely neglected [32], despite their known vulnerability to FMD.

Subclinical infections can occur in small ruminants and may serve as unnoticed reservoirs of the virus, contributing to silent transmission within farms and livestock markets [10, 43]. In Uganda, this risk could be elevated considering the common practice of cograzing cattle with small ruminants. A recent retrospective analysis of global FMD surveillance data (1958–2023) submitted to the World Reference Laboratory for FMD highlighted the understudied epidemiological role of small ruminants, largely due to the subclinical nature of infections in these species and the limited surveillance focus compared to large ruminants [44]. The study highlighted the potential involvement of small ruminants in the maintenance of FMDV and emphasized the importance of including them in FMD surveillance and control strategies, particularly in endemic settings such as Uganda. Studies on small ruminants in Uganda are limited as indicated in a recent review of the epidemiology of FMD in Uganda [32], with only two small scope studies published more than a decade ago [45, 46].

Despite Uganda’s endemic FMD status and the widespread practice of mixed‐species livestock farming, the epidemiological role of goats remains poorly characterized. The potential for goats to have subclinical infections and to contribute to silent FMD transmission highlights a critical gap in national surveillance and vaccination strategies. Understanding the extent of FMDV exposure in goats and the factors that drive infection risk is essential for designing evidence‐based FMD control programs in endemic settings. This study aimed to determine the seroprevalence of FMDV antibodies in goats and to identify the farm‐level and animal‐level risk factors associated with exposure in selected high‐risk districts of Uganda.

## 2. Materials and Methods

### 2.1. Ethical Considerations

This study was conducted in accordance with ethical approvals obtained from the University of Surrey, UK (NASPA Reference: NASPA‐2223‐08), and the Makerere University School of Veterinary Medicine, Uganda (SVAR‐IACUC 165/2023).

### 2.2. Study Design, Population, and Area

**Figure 1:**
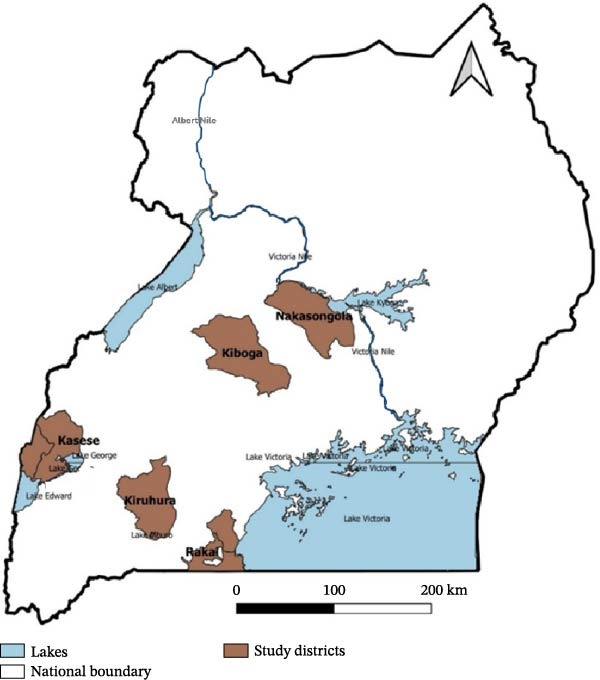
Location of the five study districts (Rakai, Kasese, Kiruhura, Kiboga, and Nakasongola).

A cross‐sectional study was conducted to investigate the seroprevalence and factors associated with FMD in goats on livestock farms across five districts in Uganda: Kasese, Kiboga, Kiruhura, Nakasongola, and Rakai (Figure 1). Serum samples were collected from goats, and farm owners were interviewed using a structured questionnaire to collect data on farm management, FMD history, disease control practices, and other related information (Supporting Information 1: File S1).

The selection of the study districts followed a risk‐based approach, prioritizing areas that had experienced FMD outbreaks within the previous 6 months as of June 2024 (Supporting Information 2: Table S1). Outbreak information was obtained from the Ministry of Agriculture, Animal Industry and Fisheries (MAAIF) outbreak records and verified through consultations with District Veterinary Officers (DVOs). One district (Kiruhura) had ongoing confirmed cases in cattle at the time of sampling, while in the remaining four districts (Kiboga, Nakasongola, Rakai, and Kasese), outbreaks had been contained but quarantine restrictions were still in place. Additional risk considerations included proximity to international borders (Kasese, Kiruhura, and Rakai), high livestock population density (Kiruhura, Nakasongola, and Kiboga), and known wildlife–livestock interfaces (Kasese and Kiruhura). This selection strategy ensured that the study was conducted in districts with diverse but theoretically important risk factors for FMD transmission in livestock.

### 2.3. Sampling

In each of the five study districts, two subcounties were selected based on recent FMD case reports from the district veterinary office. Within each subcounty, eight farms that owned goats were selected with the help of DVO. A combination of purposive sampling, targeting farms affected by recent outbreaks, and convenience sampling to include farms that were easier to access due to terrain and distance, was applied. A minimum of 10 goats were targeted for sampling on each farm. On each farm, goats were randomly selected to ensure a mix of age groups and sex. Only goats aged 5 months and above with no history of vaccination were sampled to avoid the influence of maternal antibodies or vaccine‐induced immunity on FMD serological results. Age was determined through dentition, farm records, or information from the farm owner. The required number of goats to be sampled was estimated using the single‐population proportion formula for cross‐sectional studies [47]: where *Z* = 1.96 for a 95% confidence level, *p* = 0.14 (expected seroprevalence, based on a previous study [45], and *e* = 0.05 (desired absolute precision). A minimum sample of 185 goats per district, and a total of 925 goats for the five districts, was estimated.

### 2.4. Data and Serum Collection

Data collection: A structured questionnaire (Supporting Information 1: File S1) was developed to collect data on farm management practices, FMD outbreak history, biosecurity measures, and livestock movement patterns. The questions were drafted based on previous FMD studies in East Africa and the literature and knowledge of Uganda’s local production systems. The questionnaire was pretested with five farmers in a nonstudy district (Kagadi) to assess clarity, appropriateness, and length. Feedback from the pretest was used to refine question wording and to improve logical flow. The final version was uploaded to Qualtrics to facilitate electronic data collection. After obtaining informed consent, the structured questionnaire was administered to farm owners through in‐person interviews.

Blood collection: Following the interview, goats were selected for blood sampling. Two field teams supervised by a veterinarian carried out both data and blood sample collection. For each goat, data on age, sex, breed, and body condition score were recorded using a standardized form (Supporting Information 3: File S2). Goats were gently restrained to minimize discomfort. Blood was collected from the jugular vein using 10 mL sterile vacutainer tubes with clot activator and 21‐gauge needles, and samples were labeled with unique identification codes (district, subcounty, farm, and animal number). Samples were allowed to clot in cool boxes for 6–12 h and subsequently centrifuged to separate serum. In subcounties/districts where centrifuges were not available, serum was separated manually using disposable pipettes and transferred into 2 mL cryovials. All sera were stored at –20°C in field freezers located at the respective District Veterinary Offices or, in a few cases, at nearby medical facilities until completion of sampling. The samples were then transported on ice to the laboratory at National Livestock Resources Research Institute (NaLIRRI), NARO, located in Gayaza, Wakiso District, Uganda. Serum samples were stored at –20°C until laboratory analysis.

### 2.5. Serological Analysis

The serum samples were analyzed using the FMDV 3ABC‐Trapping ELISA kit (IZSLER, Brescia, Italy) to detect antibodies against the nonstructural protein (NSP) 3ABC of FMDV, which are indicative of past or current infection regardless of vaccination status. Test and control sera were diluted 1:100 in ELISA buffer (PBS‐Tween with skimmed milk and bacterial sonicate), incubated in wells precoated with anti‐3ABC monoclonal antibodies, and then washed to remove unbound material. An antiruminant IgG peroxidase–conjugated monoclonal antibody was added, followed by a TMB substrate, and the reaction was stopped after 20 min with sulfuric acid. Optical densities (ODs) were read at 450 nm. The net OD for each sample was calculated by subtracting the OD of the well without antigen from that of the well with antigen. To normalize interassay variation, the percentage positivity (PP%) of each sample was computed as (net OD of test sample/net OD of positive control serum) × 100. A test run was considered valid if the positive control serum had a net OD > 1.0, the weak positive control serum had a PP% > 10% (typically < 50%), and the negative control serum had a PP% < 10% (typically < 5%). Samples with PP% ≥ 10% were classified as seropositive, while those with PP% < 10% were considered seronegative. Samples producing strong reactions in both antigen‐coated and uncoated wells or those with borderline PP% values were interpreted with caution and, if necessary, retested to confirm results.

### 2.6. Data Management and Analysis

Laboratory data from ELISA testing, along with individual animal biodata (species, breed, sex, age, and origin) collected during sampling, were entered into Microsoft Excel. Questionnaire data were exported from Qualtrics. All personal identifiers, including names and contacts, were removed, and unique farm and sample codes were assigned to ensure confidentiality. Data cleaning was conducted in Excel to identify and address missing values and inconsistencies. The questionnaire data were merged with the laboratory dataset to create a unified dataset. The combined dataset was coded and imported into IBM SPSS Statistics (Version 29.0.1.0; IBM Corp., Armonk, NY, USA) for statistical analysis.

Descriptive statistics were used to summarize the data, including estimates of FMD seroprevalence at animal, farm, and district levels. Following the descriptive analysis, generalized linear mixed‐effects models (GLMMs) with a binomial distribution and logit link were used to explore associations between each independent variable and the binary outcome, FMD seropositivity.

Initially, univariate analysis was performed. Independent variables with a *p*‐value < 0.25 in univariate analysis were considered for multivariable analysis. Multicollinearity among candidate categorical variables was assessed using Cramér’s *V* coefficient interpreted as follows: weak association ≤ 0.2, moderate association = 0.2–0.6, strong association ≥ 0.6. Model selection was based on Akaike’s Information Criterion (AIC), with lower values indicating better fit, Nagelkerke’s *R* ^2^ for explanatory power, and the Hosmer–Lemeshow test for goodness‐of‐fit. For each model, adjusted odds ratios (ORs) with 95% confidence intervals (CIs) were reported, along with Nagelkerke’s *R* ^2^ and classification accuracy. A *p*‐value ≤ 0.05 was considered statistically significant.

Qualitative data related to FMD control challenges were analyzed using thematic analysis, following the six‐phase framework [48]. This process involved familiarization with the data; generation of initial codes; identification of themes; reviewing, defining, and naming the themes; and producing the final report. Thematic development was conducted iteratively to ensure analytical clarity. These findings from this analysis were used to provide contextual depth to the interpretation of quantitative results.

## 3. Results

### 3.1. Descriptive Analyses

#### 3.1.1. Animal Level Characteristics of Sampled Goats

A total of 832 goats were sampled from 80 farms. Due to field logistics and variability in herd sizes, the final sample obtained (*n* = 832) was less than the estimated sample size (*n* = 925). The goats were sampled from five districts located within Uganda’s cattle corridor: Kasese (19.3%, 160/832), Kiboga (18.5%, 154/832), Kiruhura (21.7%, 180/832), Nakasongola (19.1%, 159/832), and Rakai (21.5%, 179/832). Most goats were local breeds (47.6%, 396/832), followed by crossbreeds (37.4%, 311/832), while 15.0% (125/832) were unidentified (Table 1). Regarding sex, 87.5% (728/832) were female, and 12.5% (104/832) were male. Most goats were mature (>1 year) (88.3%, 735/832), with only 11.7% (97/832) being young (≤1 year). The body condition score was reported as good (>2.5) in 48.7% (405/832) of the goats, poor (≤2.5) in 3.7% (31/832), and not clearly identified in 47.6% (396/832).

**Table 1:** Animal‐level characteristics of sampled goats.

Variable	Number of goats (%)
Breed (*n* = 832)	
Local	396 (47.60%)
Crossbreed	311 (37.38%)
Unidentified	125 (15.02%)
Sex (*n* = 832)	
Female	728 (87.50%)
Male	104 (12.50%)
Age (*n* = 832)	
Mature (>1 year)	735 (88.34%)
Young (≤1 year)	97 (11.66%)
Body condition score (*n* = 832)	
Good	405 (48.68%)
Poor (≤2.5)	31 (3.73%)
Unidentified (>2.5)	396 (47.60%)
District of origin (*n* = 832)	
Kasese	160 (19.25%)
Kiboga	154 (18.53%)
Kiruhura	180 (21.66%)
Nakasongola	159 (19.13%)
Rakai	179 (21.54%)
Goats by region of Uganda (*n* = 832)	
Central	492 (59.21%)
Western	340 (40.91%)
Goats by subcounty (*n* = 832)	
Akayanja (Kiruhura)	90 (10.83%)
Nyakashashara (Kiruhura)	90 (10.83%)
Greater Kibanda (Rakai)	99 (11.91%)
Kacheera (Rakai)	80 (9.63%0
Kapeke (Kiboga)	115 (13.84%)
Kayera (Kiboga)	39 (4.69%)
Kitswamba (Kasese)	80 (9.63%0
Muhokya TC (Kasese)	80 (9.63%)
Nabiswera (Nakasongola)	79 (9.50%)
Nakitoma (Nakasongola)	80 (9.63%)

#### 3.1.2. Farm‐Level Factors Related to FMD Occurrence and History

Data on the history of the occurrence of FMD varied across the different farms. While 37.5% (30/80) of farms reported no history of FMD, others had outbreaks: in the past 1–2 years (11.3%, 9/80), less than 6 months (20.0%, 16/80), or less than 3 months (11.3%, 9/80) (Table 2). Majority of farmers (73.8%, 48/65) observed clinical signs in their livestock during FMD outbreaks, while 16.9% (11/65) did not observe any signs, and 9.2% (6/65) could not recall. Among those who reported clinical cases, cattle were the most affected species (79.2%, 38/48). Observation of clinical cases was also reported for cattle and goats (2.6%, 1/48), cattle and pigs (2.6%, 1/48), cattle and sheep (6.2%, 3/48), and goats only (4.2%, 2/48). Among the 48 farmers who observed clinical signs during FMD outbreaks, visible lesions and salivation were the most reported signs. Approximately 17.5% (14/48) of farmers reported all six classical signs of FMD, that is, fever, lameness, loss of appetite, blisters, wounds, and salivation. Over 43.8% (21/48) observed both systemic signs (lameness + lesions/salivation ± appetite/fever) and lesion‐related signs, while very few (1.3%, 1/48) identified systemic signs alone. Neighboring farms affected by FMD were reported by 80.5% (62/77) of the farms, with 16.1% (10/62), 48.4% (30/62), and 24.2% (15/62) reporting neighboring farms being affected within the last 3 months, 6 months, and 6–12 months, respectively.

**Table 2:** Farm‐level factors related to FMD occurrence and history.

Variable	Number of farms (%)
FMD history at the farm (*n* = 80)	
<3 months ago	9 (11.3%)
<6 months ago	7 (8.8%)
>6–12 months ago	7 (8.8%)
>1–2 years ago	9 (11.3%)
>2 years ago	18 (22.5%)
Never	30 (37.5%)
Last FMD case at the farm (*n* = 79)	
10 years	1 (1.3%)
6–12 months ago	7 (8.9%)
Greater than 1 year to 2 years ago	8 (10.1%)
Less than 3 months ago	8 (10.1%)
Less than 6 months ago	17 (21.5%)
Never	30 (38.0%)
Over 2 years ago	8 (10.1%)
Did you observe any clinical signs during outbreak (*n* = 65)	
Yes	48 (73.8%)
No	11 (16.9%)
Do not know or do not remember	6 (9.2%)
Which livestock species at the farm showed clinical signs (*n* = 48)	
Cattle	38 (79.2%)
Cattle and goat	1 (2.6%)
Cattle, goat, and pig	1 (2.6%)
Cattle and sheep	3 (6.2%)
Cattle, sheep, and goat	3 (6.2%0
Goat	2 (4.2%)
Clinical signs observed during FMD outbreaks (*n* = 48)	
All six classical signs (fever, lameness, loss of appetite, blisters, wounds, and salivation)	14 (17.5%)
Visible lesions + systemic signs (e.g., lameness + lesions/salivation ± appetite/fever)	21 (43.8%)
Visible lesions only (blisters, wounds, and salivation)	3 (3.8%)
Systemic signs only (fever, lameness, and loss of appetite)	1 (1.3%)
Other sign combinations	5 (6.3%)
Did neighbors report/affected by FMD? (*n* = 77)	
Yes	62 (80.5%)
No	10 (13.0%)
I do not know or not sure	5 (6.5%)
When did neighbors report FMD (*n* = 62)	
6–12 months ago	15 (24.2%)
Greater than 1–2 years ago	7 (11.3%)
Less than 3 months ago	10 (16.1%)
Less than 6 months ago	1. (48.4%)

#### 3.1.3. Farm‐Level Factors That May Influence Exposure Risk to FMD

Most farms (79.8%, 63/79) kept cattle, sheep, or goats, while 12.7% (10/79) kept only sheep or goats. Pigs were kept by only 5.3% (4/79) of farms (Table 3). Large goat herds (>30 goats) were recorded (75.6%, 59/78), whereas sheep were more frequently kept in small herds of 1–15 (47.2%, 17/36). The cattle breeds kept were mainly crossbred (38.2%, 26/68) or a mix of local and crossbreeds (35.3%, 24/68). Large cattle herds (>50 cattle) were reported by a third of farms that kept local cattle breeds (32.4%, 11/34), by 62.5% (25/40) of farms that kept with crossbreed cattle, and by 61.4% (35/57) of farms that kept a mix of local and crossbreeds. Moving livestock out of the farm in search for water or pasture was reported by 46.7% (35/75) of farms, with daily movement being the most common (57.6%, 19/33).

**Table 3:** Frequency of farm‐level factors that may influence exposure risk to FMD.

Variable/factor	Number of farms (%)
Livestock type kept at the farm (*n* = 79)	
Cattle	1 (1.3%)
Cattle, sheep, or goats	63 (79.8%)
Cattle, sheep, goats, and pigs	3 (3.8%)
Sheep or goats	10 (12.7%)
Sheep, goats and pigs	1 (1.3%)
Type of cattle breeds kept at the farm (*n* = 68)	
Crossbreed	26 (38.2%)
Local breed	14 (20.6%)
Local breed and crossbreed	24 (35.3%)
Local breed and pure exotic	4 (5.9%)
Number of local breeds of cattle kept (*n* = 34)	
Large herd (over 50)	11 (32.4%)
Medium herd (21–50)	11 (32.4%)
Small herd (1–20)	12 (35.3%)
Number of crossbreeds of cattle kept (*n* = 40)	
Large herds (over 50)	25 (62.5%)
Medium herd (21–50)	9 (22.5%)
Small herd (1–20)	6 (15.0%)
Distance to the nearest farm (*n* = 74)	
1–5 km	26 (35.1%)
6–10 km	3 (4.1%)
Do not know	1 (1.1%)
Less than 1 km	41 (55.4%)
Over 10 km	3 (4.1%)
Total number of cattle at the farm (*n* = 57)	
Large herd (over 50)	35 (61.4%)
Medium size (21–50)	15 (26.3%)
Small herd (1–20)	7 (12.3%)
Sheep number kept on farm (*n* = 36)	
Large herd (over 30)	7 (19.4%)
Medium herd (16–30)	12 (33.3%)
Small herd (1–15)	17 (47.2%)
Goat number kept on farm (*n* = 78)	
Large herd (over 30)	59 (75.6%)
Medium herd (16–30)	17 (21.8%)
Small herd (1–15)	2 (2.6%)
Missing	754 (90.6%)
Total number of small ruminants on farm (*n* = 78)	
Large herd (over 30)	65 (83.3%)
Medium herd (16–30)	11 (14.1%)
Small herd (1–15)	2 (2.6%)
Farm own pigs (*n* = 76)	
No	72 (94.7%)
Yes	4 (5.3%)
Bought in any animals in past 12 months (*n* = 80)	
No	32 (40)
Yes	48 (60)
Number of animals bought in recently (*n* = 48)	
Large number bought (16–30)	6 (12.5)
Medium number bought (6–15)	18 (37.5)
Small number bought (1–5)	24 (50)
Wildlife–livestock interaction occurred in the past 2 years (*n* = 69)	
No	37 (53.6%)
Yes	32 (46.4%)
Do you move your animals for some reasons (*n* = 75)	
No	40 (53.3%)
Yes	35 (46.7%)
How frequently do you move your animals (*n* = 33)	
Daily	19 (57.6%)
Seasonally	11 (33.3%)
Monthly	1 (3.0%)
Others	2 (6.1%)
Do you move animals across boarder (*n* = 17)	
No	10 (58.8%)
Yes	7 (41.2%)
Do you share grazing land (*n* = 7)	
No	3 (42.9%)
Yes	4 (57.1%)
How frequently o you share grazing land (*n* = 4)	
Daily	2 (50.0%)
Seasonally	2 (50.0%)
Do you share grazing land in the dry season (*n* = 72)	
No	36 (50.0%)
Yes	36 (50.0%)
How long the distance you move animals in dry season (*n* = 35)	
Less than 5 km	19 (54.3%)
5–10 km	13 (37.1%)
More than 10 km	3 (8.6%)
Is your farm located near an international border (*n* = 75)	
No	49 (65.3%)
Yes	26 (34.7%)
Is your farm located near the national park (*n* = 75)	
No	38 (50.7%)
Yes	37 (49.3%)
What is the distance to the nearest NP from your farm (*n* = 36)	
Less than 5 km	20 (55.6%)
5–10 km	7 (19.4%)
More than 10 km	6 (16.7%)
Do not know	3 (8.3%)

Cross‐border animal movement was reported by 41.2% (7/17) of respondents. About 50.0% (36/72) of farms reported sharing grazing land in the dry season, with most movements occurring within a 5 km range (54.3%, 19/35). Wildlife–livestock interaction was noted on 46.4% (32/69) of farms, while 49.3% (37/75) were located near a national park, with 55.6% (20/36) being within 5 km of the park. The buying of animals into the farm was common, with 60.0% (48/80) of farms purchasing animals in the past 12 months. Among these, most farms bought a small number (1–5 animals: 50.0%, 24/48), while 12.5% (6/48) purchased between 16 and 30 animals. Farms were often in proximity to other livestock holdings, with 55.4% (41/74) reporting distances of less than 1 km to the nearest farm.

#### 3.1.4. Farm Level/Factors Related to Management Practices

Most cattle farms used either a free‐range system 35.3% (24/68) or a paddock‐based system 27.9% (19/68) (Table 4). Goats were predominantly raised under extensive/traditional systems 75.6% (59/78), with fewer managed under semi‐intensive systems 23.1% (18/78). Pig farming was evenly split between intensive/commercial 50.0% (2/4) and semi‐intensive 50.0% (2/4) systems. Silage use was minimal, with most farms not using silage for cattle 87.7% (57/65) or small ruminants 91.7% (66/72). Half of the farms were fenced 50.0% (40/80), but only 43.4% (33/76) of fences were effective at preventing intrusion by other animals. Footbaths were present on 12.7% (10/79) of farms, and only half of these 50.0% (5/10) were actively being used.

**Table 4:** Frequency of farm‐level factors related to management practices.

Variable/factor	Number of farms (%)
Farming experience (*n* = 75)	
10 to 15 years	7 (9.30%)
15 to 20 years	8 (10.70%)
5 to 10 years	16 (21.30%)
More than 20 years	44 (58.70%)
What type of cattle farming system do you use (*n* = 68)	
Free‐range	24 (35.3%)
Paddock	19 (27.9%)
Pastoral system	3 (4.4%)
Ranching	2 (2.9%)
Others	20 (29.4%)
What type of goat farming system do you use (*n* = 78)	
Extensive/traditional system	59 (75.6%)
Semi‐intensive system	18 (23.1%)
Intensive system	1 (1.3%)
What type of pig farming system do you use (*n* = 4)	
Intensive/commercial system	2 (50.0%)
Semi‐intensive system	2 (50.0%)
Do you make silage for cattle (*n* = 65)	
No	57 (87.7%)
Yes	8 (12.3%)
Do you make silage for small ruminants (*n* = 72)	
No	66 (91.7%)
Yes	6 (8.3%)
Do you feed pigs on kitchen leftover (*n* = 3)	
No	2 (66.7%)
Yes	1 (33.3%)
Is your farm fenced (*n* = 80)	
No	40 (50.0%)
Yes	40 (50.0%)
Can the fence prevent animal intrusion (*n* = 76)	
No	43 (56.6%)
Yes	33 (43.4%)
Does the farm have gate (*n* = 78)	
No	48 (61.5%)
Yes	30 (38.5%)
Does the farm have footbath (*n* = 79)	
No	69 (87.3%)
Yes	10 (12.7%)
Is the footbath being used (*n* = 10)	
No	5 (50.0%)
Yes	5 (50.0%)
What FMD prevention strategies do you use (*n* = 80)	
Vaccination	11 (13.8%)
Regular veterinary check‐ups	5 (6.3%)
Isolation of new animals	14 (17.5%)
Combination of strategies	50 (62.5%)
When did you last vaccinate against FMD (*n* = 68)	
Less than 6 months ago	47 (69.1%)
1 year ago	10 (14.7%)
1.5 years ago	3 (4.4%)
2 years ago	3 (4.4%)
Never	3 (4.4%)
I do not know	2 (2.9%)
How frequently do you vaccinate against FMD (*n* = 68)	
Once a year	24 (35.3%)
Twice a year	23 (33.8%)
Only during outbreaks	19 (27.9%)
Others	2 (2.9%)
What was the source of the FMD vaccine (*n* = 70)	
Government	64 (91.4%)
Private vets	1 (1.4%)
Government and private vets	3 (4.3%)
Others	2 (2.9%)
Have you ever received any FMD training (*n* = 80)	
No	28 (35.0%)
Yes	52 (65.0%)
How frequently have you received FMD training (*n* = 50)	
Twice a year	27 (54.0%)
During outbreaks	13 (26.0%)
As the need may arise	4 (8.0%)
Others	6 (12.0%)
What was the source of FMD training (*n* = 48)	
Government Veterinary Officer	20 (41.7%)
Vets and fellow farmers	9 (18.8%)
Veterinary extension workers	4 (8.3%)
Others	15 (31.3%)

For FMD prevention, most farmers (62.5%, 50/80) reported using a combination of strategies, including vaccination, regular checks, and isolation of new animals (Table 4). Vaccination alone was used by 13.8% (11/80) of farms. Most farms reported vaccinating within the last 6 months 69.1% (47/68), following either annual 35.3% (24/68) or biannual 33.8% (23/68) vaccination schedules. The majority sourced vaccines from the government 91.4% (64/70). Regarding FMD training, 65.0% (52/80) of farms had received training. Among those trained, most received training twice a year 54.0% (27/50) or during outbreak periods 26.0% (13/50). Training was mainly provided by government veterinary officers 41.7% (20/48) or by a combination of veterinarians and fellow farmers 18.8% (9/48).

#### 3.1.5. FMD Seroprevalence at Animal, Farm, and District Levels

**Figure 2:**
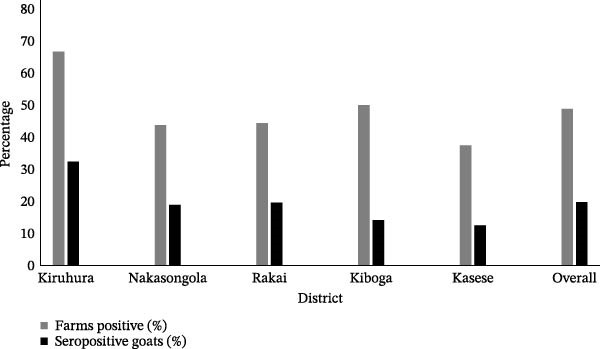
Proportion of FMD positive farms and seropositive goats by district.

The details of animal‐level, farm‐level, and district‐level seroprevalence are provided in Supporting Information 2: Table S2. The overall animal‐level seroprevalence was 19.8% (165/832; 95% CI: 17.26%–22.68%), with 48.8% of farms (39/80) having at least one seropositive goat. Farm‐level positivity ranged from 0% to 100%. Seroprevalence varied significantly between districts, ranging from 12.5% to 32.4%. A summary of farm‐ and district‐level seroprevalence is provided in Table 5 and visualized in Figure 2. Kiruhura district had the highest seroprevalence (32.4%, 58/179), with Akayanja (Kanyaryeru) subcounty representing a major hotspot, where seven of nine sampled farms (77.8%) had seropositive goats, including three farms with seroprevalence between 70% and 80%. Nyakashashara subcounty of Kiruhura district had five of nine farms (55.6%) testing positive, though only one farm had 50% prevalence. Only four farms in the Kiruhura district were negative.

**Table 5:** Summary of district‐level and farm‐level seroprevalence of FMD in goat (details provided in Supporting Information 2: Table S2).

District	Total farms sampled	Farms with ≥1 positive goat	Proportion of farms positive	Total goats tested	Number seropositive goats	Proportion of seropositive goats % (CI)	Key subcounty findings
Kiruhura	18	12	66.7%	179	58	32.4% (25.98–39.57)	Akayanja: seven/nine farms positive (70%–80% positivity in three farms)
Nakasongola	16	7	43.8%	159	30	18.9% (13.55–25.66)	Nakitoma: seven/eight farms positive (one farm at 80% positivity)
Rakai	18	8	44.4%	179	35	19.6% (14.41–25.98)	Kacheera: one farm at 100% positivity; Greater Kibanda: six/10 farms positive
Kiboga	12	6	50.0%	155	22	14.2% (9.56–20.56)	Kapeke: five/nine farms positive (highest: 46.7% positivity)
Kasese	16	6	37.5%	160	20	12.5% (8.24–18.52)	Kitswamba: six/eight farms positive; Muhokya TC: zero/eight farms positive
Overall	80	39	48.8%	832	165	19.8% (17.26–22.68)	Nearly half of the farms sampled had FMD seropositive goats

Rakai district exhibited variable seroprevalence, with an overall prevalence of 19.6% (35/179). In Kacheera subcounty, a single farm showed 100% positivity (10/10 goats), although five of the eight farms in this subcounty had no seropositive animals. In Greater Kibanda subcounty, six of 10 farms (60%) were positive, including two farms with seroprevalence between 44.4% and 50%. Nakasongola district had an overall seroprevalence of 18.9% (30/159), with Nakitoma subcounty as the focal point, where five of eight farms (62.5%) were positive, including two farms with prevalence rates of 50% and 80%. In contrast, Nabiswera subcounty showed minimal prevalence, with only one of eight farms testing positive. Kiboga district had an overall seroprevalence of 14.2% (22/155), indicating moderate transmission, with Kapeke subcounty having five of nine farms (55.6%) positive, including one farm with a prevalence of 46.7% prevalence. The Kasese district had the lowest overall seroprevalence (12.5%, 20/160), with all positive cases concentrated in the Kitswamba subcounty, where six of eight farms were positive. Notably, Muhokya TC subcounty showed no seropositive goats across all eight farms.

### 3.2. Univariable Logistic Regression: Risk Factors Associated With FMD Seropositivity in Goats

The relationship between FMD seropositivity and potential risk factors was first examined using univariable binary logistic regression. Key findings for the variables with *p* ≤ 0.25 are summarized below, and details are presented in Supporting Information 2: Table S3.

#### 3.2.1. Animal‐Level Factors

Univariable analysis of individual animal characteristics (Supporting Information 2: Table S3) showed that breed was associated with FMD seropositivity (*p* = 0.027). Goats of crossbreed origin had lower odds of seropositivity (OR = 0.66, CI = 0.45–0.95) compared to local breeds.

#### 3.2.2. Farm‐Level Factors Related to FMD Occurrence and History

The district of origin was associated with FMD seropositivity. Goats from Kiruhura district had higher odds (OR = 2.90, CI = 1.67–5.02, *p* ≤ 0.001) compared to those from Kiboga. Farms with a history of FMD in the previous 2 years had higher odds of having seropositivity goats compared to farms with no history of FMD (reference): <6 months ago: OR = 3.22, CI = 2.05–5.08, *p* ≤ 0.001; 6–12 months ago: OR = 2.83, CI = 1.47–5.44, and *p* = 0.002; 1 year ago: OR = 3.03, CI = 1.86–4.93, *p* < 0.001. Similarly, the time since the last FMD case on the farm was associated with seropositivity. Farms that reported affected by FMD < 3 months ago (OR = 4.83, CI = 2.76–8.45, *p* ≤ 0.001), <6 months ago (OR = 2.17, CI = 1.31–3.59, *p* = 0.003), 6–12 months ago (OR = 2.57, CI = 1.34–4.90, *p* = 0.004), and 1–2 years ago (OR = 2.19, CI = 1.16–4.16, *p* = 0.016) all had higher odds compared to farms that had never had a case (reference). Observation of clinical signs of FMD during outbreaks on the farm was associated with higher odds of seropositivity (OR = 3.50, CI = 1.82–6.72, *p* ≤ 0.001) compared to farms that reported no clinical signs (reference). Farms located next to those that had experienced FMD outbreak in the past 3 months had higher odds of seropositivity (OR = 3.43, CI = 1.60–7.33, *p* ≤ 0.001) compared to farms whose neighbors’ most recent FMD outbreak occurred more than 1 to 2 years ago (reference group). Similarly, having a neighboring farm that experienced an FMD outbreak at any time within the past 2 years was also associated with increased odds of goats being exposed to FMDV (OR = 2.40, CI = 1.31–4.39, *p* = 0.005) compared to farms with neighbors that had no outbreaks.

#### 3.2.3. Farm‐Level Factors Related to FMD Exposure Risk

Farming experience showed an association with seropositivity. Farmers with 10–15 years of experience (OR = 0.14, CI = 0.04–0.45, *p* ≤ 0.001) and 5–10 (OR = 0.57, CI = 0.35–0.93, *p* = 0.024) had lower odds compared to farmers with more than 20 years of experience (reference). Wildlife interaction within the past 2 years was associated with seropositivity (OR = 1.95, CI = 1.34–2.85, *p* ≤ 0.001) compared to no reported interaction (reference). Farms located near a national park had higher odds (OR = 2.09, CI = 1.46–2.98, *p* ≤ 0.001) compared to those not near a park (reference). Distance to the nearest park also showed an association: farms within 5 km (OR = 0.40, CI = 0.21–0.73, *p* = 0.003), and those uncertain about the distance (OR = 0.22, CI = 0.07–0.70, *p* ≤ 0.010) had lower odds compared to farms more than 10 km away (reference). Farms located near an international border had lower odds (OR = 0.66, CI = 0.45–0.99, *p* = 0.042) compared to those not near a border (reference).

#### 3.2.4. Farm Management Practices

Large sheep flocks (>30 head) were associated with higher odds of seropositivity (OR = 3.90, CI = 2.04–7.44, *p* ≤ 0.001) compared to small flocks (1–15 head, reference). Recent purchase of animals was associated with lower odds (OR = 0.62, CI = 0.44–0.87, *p* = 0.005) compared to no recent purchases. Among purchased cattle, medium numbers (6–15 head) were associated with higher odds (OR = 1.96, CI = 1.20–3.20, *p* = 0.007) compared to small numbers (1–5 head, reference). Monthly movement of animals was associated with markedly higher odds (OR = 36.00, CI = 4.28–302.80, *p* ≤ 0.001) compared to other movement patterns (reference: “Others”). Absence of cattle silage production was associated with higher odds (OR = 1.96, CI = 1.01–3.80, *p* = 0.046) compared to farms that made silage (reference). Receiving FMD‐related training was associated with lower odds (OR = 0.64, CI = 0.44–0.94, *p* = 0.022) compared to those without training (reference).

### 3.3. Multivariable Logistic Regression: Factors Associated With FMD Seropositivity in Goats

Variables associated with FMD seropositivity in goats at a significance level of *p*  < 0.25 in the univariable analysis (Supporting Information 2: Table S3) were considered as candidates for the multivariable model. Prior to model building, candidate categorical variables were assessed for multicollinearity. The results of the multicollinearity assessment are presented in Supporting Information 2: Table S4. When a strong association was identified between two variables, one of them was excluded from further analysis. Following multicollinearity assessment, five multivariable logistic regression models were developed and compared (Supporting Information 2: Table S5).

Model 1 was selected as the best‐performing model, demonstrating the highest explanatory power (Nagelkerke *R* ^2^ = 0.224), the lowest AIC value (607.29), and a better model fit (Hosmer–Lemeshow test, *p* = 0.809) (Table 6). The model included the largest sample size (*N* = 657/832) and achieved an overall classification accuracy of 79%. In this final model, several predictors were significantly associated with FMD seropositivity. Farms located near a national park had markedly higher odds of FMD seropositivity (OR = 14.37; 95% CI: 3.80–54.29) compared to farms far from the national park. A recent history of FMD on the farm was associated with FMD seropositivity, particularly for a history of outbreaks occurring within the past 6 months (OR = 6.63; 95% CI: 3.58–12.28). The breed of goats and the district of origin were also significant predictors. Crossbred goats had significantly lower odds of FMD seropositivity compared to local breeds (OR = 0.51; 95% CI: 0.31–0.86). Similarly, farms in Kasese, Kiruhura, and Nakasongola districts had significantly lower odds of FMD seropositivity compared to the reference district, Kiboga.

**Table 6:** Multivariable logistic regression analysis.

Model 1	Exp(B)	95% CI for EXP(B)		*p*‐Value
		Lower	Upper	
Crossbred goats	0.514	0.308	0.856	0.011
Local breed goats (ref)				
Kasese district	0.009	0.002	0.050	≤0.001
Kiruhura district	0.052	0.011	0.248	≤0.001
Nakasongola district	0.093	0.027	0.323	≤0.001
Rakai district	0.389	0.143	1.056	0.064
Kiboga district (REF)				
Farm located near national park (yes)	14.371	3.804	54.286	≤0.001
Farm located near national park (no) (REF)				
FMD hist (<6 months ago)	6.628	3.578	12.278	≤0.001
FMD hist (6–12 months ago)	1.543	0.480	4.961	0.467
FMD hist (>1 year ago)	2.189	1.218	3.933	0.009
FMD hist (never had FMD) (REF)				

### 3.4. Thematic Analysis of FMD Control Challenges Reported by the Farmers

A summary of the reported FMD control challenges faced by farmers is presented in Table 7. The most frequently reported issues by farmers were inadequate and delayed vaccine supply, communal grazing that promotes herd mixing, and uncontrolled animal movement, particularly during quarantine. Farmers also cited the high cost of vaccination, irregular vaccination, often limited to outbreak periods, and tick infestation linked to shared grazing areas. Additional challenges included shared watering points, violations of quarantine regulations, economic losses during outbreaks, such as reduced milk and animal sales and increased costs of living, lack of awareness of FMD transmission and control, and limited grazing land. The less frequently reported challenges included ineffective vaccines, limited access to disinfectants, and late recognition of FMD clinical signs.

**Table 7:** Thematic analysis of FMD control challenges reported by the farmers.

Theme	Description	Frequency of mention	Example of challenges mentioned
Lack of vaccines	Government‐provided vaccines are inadequate and delayed	15	“Vaccines are not available”; “Government delays in vaccination”
Animal mixing and communal grazing	Communal grazing increases disease transmission	15	“Animals mix in maize fields post‐harvest”
Uncontrolled animal movement	Illegal movement, especially during quarantine, spreads FMD	15	“Animals moved illegally under quarantine”
High cost of vaccination	Vaccines and veterinary services are unaffordable	10	“It’s costly to buy the vaccine”; “Animal vaccination price is high”
Irregular vaccinations	Vaccination is mainly done during outbreaks rather than preventively	10	“Vaccination only happens when there is an outbreak”
Tick infestation	Linked to communal grazing and poor pasture management	10	“Ticks infest animals grazing together”
Shared watering points	Government valley dams serve as FMD transmission hotspots	5	“Animals share watering points”
Quarantine violations	Farmers often violate restrictions, undermining control efforts	5	“Animals moved despite quarantine”
Economic losses	Reduced milk and animal sales, increased costs of living	5	“No milk or animal sales during outbreaks”
Lack of training	Farmers lack awareness of FMD transmission and control	5	“No training on FMD control measures”
Poor pasture management	Limited grazing land forces animals to mix, increasing risk	5	“Pastures are scarce, especially in the dry season”
Fake or ineffective vaccines	Some farmers report receiving ineffective vaccines	3	“Fake vaccines”; “Ineffective vaccines”
Lack of disinfectants	Limited access to disinfectants hampers disease control	3	“No disinfectants available”
Early detection issues	Late recognition of FMD symptoms delays intervention	2	“Delayed detection of disease signs”

## 4. Discussion

### 4.1. Seroprevalence and the Role of Goats in FMD Epidemiology

This study investigated FMD seroprevalence and the factors associated with FMD in goats across five districts in Uganda using a risk‐based sampling approach and provides a systematic assessment of FMD epidemiology in goats in the country. The overall animal‐level seroprevalence of 19.8% and farm‐level prevalence of 48.8% in the current study are higher compared to previous studies. A previous study reported a prevalence of 14% in goats sampled as part of surveillance activities [45], while another study reported a prevalence of 17% in goats sampled during nonoutbreak periods [46]. However, it is important to note that both previous studies were limited by small sample size and less rigorous sampling designs. Comparisons with endemic regions elsewhere in Africa show variation in prevalence. In Ethiopia, a seroprevalence of 7% was reported [49], 10.2% in Nigeria, 22.5% in Kenya [50], and 14.1% in Sudan [51]. The observed country variation in prevalence may be due to differences in epidemiological context, host populations, timing relative to outbreaks, and sampling strategy.

Although the reported animal‐level seroprevalence in goats in the current study is lower compared to that observed in cattle, the high farm‐level prevalence and presence of farms with 100% seropositive goats indicate substantial exposure of goats and a high likelihood of farm‐level infection. In cattle, animal and herd level prevalences of 50%–77% [35, 46, 49] and 63%–86.7% [35, 38], respectively, have been reported in Uganda. The subclinical infection in goats may lead to silent maintenance hosts, facilitating undetected viral persistence and spread within mixed herds and through livestock movements. A retrospective analysis of 32,802 samples submitted to the World Reference Laboratory for FMD (1958–2023), including 1972 small ruminants, reported 30% positivity and highlighted challenges in detecting infection in these species [44]. Collectively, our findings suggest that goats may play a significant role in the epidemiology and maintenance of FMD than currently recognized.

In Uganda, as in many resource‐constrained endemic settings, small ruminants are frequently overlooked and/or excluded from FMD surveillance and vaccination programs. FMD infection in goats has been described as mild or subclinical. Earlier studies indicate that approximately a quarter of infected small ruminants may not develop any vesicular lesions, while about 20% may develop only a single, subtle lesion [50–52]. Such subtle manifestations make detection challenging under field conditions, where routine diagnostic capacity is limited, potentially creating a false impression among farmers and surveillance teams that goats are uninfected. Consistent with previous reports [51], farmers in the current study described lameness, occasional vesicular lesions, fever, and other mild signs during outbreaks, which may affect productivity, including milk yield and growth. In the present study, 79% of farms reported cograzing of goats with cattle, which offers an opportunity for exposure of susceptible species to FMDV. In mixed‐species farms, vaccinating cattle alone creates immunity gaps in goats, potentially allowing continued virus circulation and transmission. These findings underscore the need to integrate small ruminants into routine FMD surveillance and vaccination programs.

### 4.2. Key Risk Factors for FMD Seropositivity in Goats

Multivariable analysis identified four factors significantly associated with FMD seropositivity in goats: outbreak history at the farm, proximity to national parks, goat breed, and district of origin. As would be expected, farms that experienced outbreaks within the previous 6 months had over six times higher odds of seropositivity compared to farms with no prior FMD history. Notably, even farms with outbreaks that occurred more than a year earlier were significantly associated with seropositivity, suggesting that once FMD is introduced, the risk can remain long after the outbreak event. This finding is consistent with the known persistence of FMDV; the virus is highly contagious and can survive for several days to weeks in the environment depending on temperature and humidity and can remain viable on fomites such as equipment, feed, and bedding [8, 10]. Studies have shown that FMDV has remarkable environmental persistence, which poses significant challenges to disease control on affected farms. The virus can persist for over 28 days in water at 20°C [53], 25 days in soil depending on humidity and temperature [53], and more than 75 days on vegetation under high humidity [54], creating prolonged indirect exposure risks on affected farms.

In Uganda, the risk of FMD persistence in the environment is amplified by mixed cattle and small ruminant herds, communal grazing, wildlife–livestock interactions in some districts, and poor farm‐level biosecurity, all of which facilitate repeated contact with contaminated environments. Additionally, different host species can establish FMDV carrier state for varying periods. For example, cattle can harbor FMDV for up to 33 months [10], sheep and goats for up to 9 months [20], and up to 5 years in African buffalo [22]. Together, the combination of environmental persistence, management factors, and prolonged host carrier states across species enables sustained virus circulation, prolongs transmission cycles, and complicates outbreak control efforts.

Proximity to national parks was a strong predictor of infection, with farms near wildlife interfaces exhibiting over 14 times higher odds of seropositivity. This finding aligns with the well‐documented role of wildlife–livestock interactions in FMD transmission [55, 56]. African buffalo (*Syncerus caffer*) are known long‐term reservoirs of FMDV, and spillover to livestock often occurs at these interfaces [55, 57–59]. In the current study, Kasese and Kiruhura districts both bordering Queen Elizabeth and Lake Mburo National Parks, respectively, reflected this heightened risk, underscoring the importance of FMD surveillance and vaccination around wildlife–livestock interfaces.

Goat breed was significantly associated with seropositivity, with local goats showing higher odds of infection compared to crossbred goats. In Uganda, however, breed distinctions are often blurred; more than 97% of the national goat population consists of indigenous types, and most crossbred goats are mixed with local stock rather than forming distinct exotic lines [60]. This makes it unlikely that genetic differences alone explain the observed association. Instead, the higher seropositivity among local goats may reflect subtle but meaningful differences in exposure patterns linked to how these animals are raised. Although management systems across farms in the study districts are broadly similar, households keeping crossbred goats tend to adopt slightly more controlled husbandry practices, such as maintaining more closed herds and less use of communal grazing, which may reduce opportunities for FMD exposure. This interpretation is supported by the finding that farms not producing cattle silage had significantly higher odds of FMD seropositivity, suggesting that farms engaging in more structured feeding and management practices may also implement better disease‐prevention measures. Breed‐related differences in FMD seroprevalence have previously been documented in cattle in Ethiopia [61, 62], although similar evidence in small ruminants is largely absent. In the context of our study and considering the highly mixed nature of goat populations in Uganda, where only 2%–3% are exotic or crossbreeds [60], the observed breed effect is more plausibly explained by differences in management systems rather than intrinsic breed susceptibility.

The district of origin was associated with FMD seropositivity, indicating spatial heterogeneity of exposure risk across Uganda. Farms in Kasese, Kiruhura, and Nakasongola had lower adjusted odds of seropositivity compared to Kiboga. Kiboga district, which showed the highest adjusted odds, is characterized by high levels of animal movement for trade and grazing, extensive communal grazing systems, recurrent outbreaks, and poor farm‐level biosecurity (including limited fencing). Previous studies in Uganda reported spatial clustering of FMD, with higher risk in areas characterized by high cattle density, low annual rainfall, and pastoral production systems, and near international borders [33, 35]. The ecological conditions and management factors vary across the districts examined in the current study and may partly explain the observed differences in seroprevalence. These conditions facilitate frequent interherd contact and increase opportunities for viral transmission. Together, these findings highlight the importance of geographically targeted, risk‐based FMD surveillance and control strategies, prioritizing districts with high livestock movement and limited biosecurity infrastructure.

### 4.3. Constraints to FMD Control

Several systemic challenges that hinder effective FMD control were reported, including inadequate vaccine supply, uncontrolled animal movement, and widespread communal grazing. Heavy reliance on government‐led vaccination programs, which provide 91.4% of all vaccines (as reported in Table 4 and evidenced in Table 7), often results in delays, shortages, and irregular vaccination campaigns with limited coverage. Although centralized programs can ensure quality oversight, they are frequently constrained by budgetary and logistical limitations. Notably, the Ugandan government reported in 2024 that it could procure vaccines for only 1.1% of the eligible livestock population annually, highlighting a substantial gap in vaccine availability and coverage [42]. FMD experts have indicated that subsidized or regulated private‐sector involvement especially in challenged endemic settings can improve access to FMD vaccines, increase uptake, and expand vaccination coverage [15, 63, 64]. Expanding such private sector participation in Uganda may enhance vaccine availability while maintaining quality standards.

Biosecurity‐related challenges were reported in the current study, particularly communal grazing and frequent animal movement. Communal grazing promotes herd mixing and facilitates FMD transmission and is common in Uganda [30] and other FMD endemic regions such as Ethiopia and Tanzania [65, 66]. Frequent animal movement for trade and seasonal grazing further increases exposure risk [67, 68]. In the current study, farmers also reported violations of quarantine measures during outbreaks, reflecting low compliance and weak enforcement, which have been documented in other endemic settings [67, 68]. The gaps in vaccination, biosecurity, and movement control likely contribute to sustained viral circulation, which is consistent with the widespread seropositivity observed in this study. Addressing these challenges will require a combination of continuous engagement with farming communities to promote compliance with vaccination, biosecurity, and movement control measures; improved coordination between veterinary authorities and local government; and stronger policy enforcement.

## 5. Conclusion

This study provides evidence of FMDV exposure in goats in Uganda and shows that small ruminants may play a substantial role in FMD epidemiology than currently reflected in national policies. The observed exposure factors, including proximity to wildlife, breed patterns, and district, indicate that goats are predisposed through management and ecological factors. These findings are important because Uganda’s current FMD control strategy focuses almost exclusively on cattle, leaving immunity gaps in the mixed herds where goats and cattle cograze. The study also identified systemic challenges, including inadequate vaccine supply, irregular vaccination campaigns, communal grazing, and weak enforcement of movement controls, all of which contribute to continued virus spread and circulation. Addressing these challenges is essential to breaking the transmission cycle and reducing the national burden of FMD. To further strengthen FMD control efforts, small ruminants should be considered for targeted surveillance and vaccination, especially in districts with recurrent outbreaks, close to wildlife–livestock interfaces, and with extensive communal grazing. Finally, improving vaccine delivery systems, including regulated private‐sector involvement, alongside stronger movement control enforcement and practical farm‐level biosecurity (e.g., controlled grazing, better fencing, and farmer sensitization), will be critical in strengthening national policy on FMD control and safeguarding the livelihoods of livestock‐dependent communities.

## Funding

This research was supported by funding from the Commonwealth Scholarship Commission (CSC), UK and the University of Surrey, UK.

## Conflicts of Interest

The authors declare no conflicts of interest.

## Supporting Information

Additional supporting information can be found online in the Supporting Information section.

## Supporting information


**Supporting Information 1** File S1: Questionnaire used to collect data on farm management, FMD history, disease control practices, and other related information.

**Supporting Information 2** Table S1: The selection criteria of the study districts following a risk‐based approach. Supporting Table S2: Animal level, farm level, and district level seroprevalence. Supporting Table S3: Results of univariable logistic regression—factors associated with FMD seropositivity. Supporting Table S4: Results of multicollinearity assessment. Supporting Table S5: Results of multivariable logistic regression analyses—seven different models evaluated.

**Supporting Information 3** File S2: Sample collection form used to identify the sample and capture animal biodata and geographical information.

## Data Availability

The data that support the findings of this study are available from the corresponding author upon reasonable request.

## References

[1] Waldmann O T. K. , Experimentelle Untersuchungen über die Pluralität des Maul-und Klauenseuchevirus, Berliner und Münchener Tierärztliche Wochenschrift. (1926) 42, 569–571.

[2] Vallée H. and Carré H. , On the Plurality of the Foot-and-Mouth Disease Virus, Comptes Rendus de l’Académie des Sciences. (1922) 174, 1498–1500.

[3] Brooksby J. B. and Rogers J. , Methods Used in Typing the Virus of Foot-and-Mouth Disease at Pirbright, Methods of Typing and Cultivation of Foot-and-Mouth Disease Virus, 1957, European Productivity Agency of the Organization of European Cooperation (OEEC), 1950–1955, Methods of Typing and Cultivation of Foot-and-Mouth Disease Virus: Project No. 208.

[4] Brooksby J. B. , The Virus of Foot-and-Mouth Disease, Advances in Virus Research. (1958) 5, 1–37, 10.1016/S0065-3527(08)60670-3, 2-s2.0-0012482026. 13508401

[5] Grubman M. J. and Baxt B. , Foot-and-Mouth Disease, Clinical Microbiology Reviews. (2004) 17, no. 2, 465–493, 10.1128/CMR.17.2.465-493.2004, 2-s2.0-2042459160. 15084510 PMC387408

[6] Jamal S. M. and Belsham G. J. , Foot-and-Mouth Disease: Past, Present and Future, 2013. 10.1186/1297-9716-44-116 PMC4028749 24308718

[7] Alexandersen S. and Mowat N. , Foot-and-Mouth Disease: Host Range and Pathogenesis, Current Topics in Microbiology and Immunology. (2005) 288, 9–42, 10.1007/3-540-27109-0_2. 15648173

[8] Alexandersen S. , Quan M. , Murphy C. , Knight J. , and Zhang Z. , Studies of Quantitative Parameters of Virus Excretion and Transmission in Pigs and Cattle Experimentally Infected with Foot-and-Mouth Disease Virus, Journal of Comparative Pathology. (2003) 129, no. 4, 268–282, 10.1016/S0021-9975(03)00045-8, 2-s2.0-0042036880. 14554125

[9] Paton D. J. , Gubbins S. , and King D. P. , Understanding the Transmission of Foot-and-Mouth Disease Virus at Different Scales, Current Opinion in Virology. (2018) 28, 85–91, 10.1016/j.coviro.2017.11.013, 2-s2.0-85037716197. 29245054

[10] Stenfeldt C. and Arzt J. , The Carrier Conundrum; A Review of Recent Advances and Persistent Gaps Regarding the Carrier State of Foot-and-Mouth Disease Virus, Pathogens. (2020) 9, no. 3, 10.3390/pathogens9030167, 167. 32121072 PMC7157498

[11] Kitching R. P. and Hughes G. J. , Clinical Variation in Foot and Mouth Disease: Sheep and Goats, Revue Scientifique et Technique de l’OIE. (2002) 21, no. 3, 505–512, 10.20506/rst.21.3.1342, 2-s2.0-0036939869. 12523691

[12] Hughes G. J. , Mioulet V. , Kitching R. P. , Woolhouse M. E. J. , Alexandersen S. , and Donaldson A. I. , Foot-and-Mouth Disease Virus Infection of Sheep: Implications for Diagnosis and Control, Veterinary Record. (2002) 150, no. 23, 724–727, 10.1136/vr.150.23.724, 2-s2.0-0037042426. 12081308

[13] Alexandersen S. , Zhang Z. , Donaldson A. I. , and Garland A. J. M. , The Pathogenesis and Diagnosis of Foot-and-Mouth Disease, Journal of Comparative Pathology. (2003) 129, no. 1, 1–36, 10.1016/S0021-9975(03)00041-0, 2-s2.0-0042805199. 12859905

[14] WOAH , Foot-and-Mouth Disease (FMD), 2025, https://www.woah.org/en/disease/foot-and-mouth-disease/.

[15] Knight-Jones T. J. D. and Rushton J. , The Economic Impacts of Foot and Mouth Disease - What Are They, How Big Are They and Where Do They Occur?, Preventive Veterinary Medicine. (2013) 112, no. 3-4, 161–173, 10.1016/j.prevetmed.2013.07.013, 2-s2.0-84886792476. 23958457 PMC3989032

[16] Al-Salihi K. A. , The Epidemiology of Foot-and-Mouth Disease Outbreaks and Its History in Iraq, Veterinary World. (2019) 12, no. 5, 706–712, 10.14202/vetworld.2019.706-712, 2-s2.0-85068867008. 31327908 PMC6584858

[17] Hayer S. S. , Ranjan R. , and Biswal J. K. , et al.Quantitative Characteristics of the Foot-and-Mouth Disease Carrier State Under Natural Conditions in India, Transboundary and Emerging Diseases. (2018) 65, no. 1, 253–260, 10.1111/tbed.12627, 2-s2.0-85014099671. 28251837

[18] Bertram M. R. , Vu L. T. , and Pauszek S. J. , et al.Lack of Transmission of Foot-and-Mouth Disease Virus From Persistently Infected Cattle to Naïve Cattle Under Field Conditions in Vietnam, Frontiers in Veterinary Science. (2018) 5, 10.3389/fvets.2018.00174. PMC6072850 30101147

[19] Burrows R. , The Persistence of Foot-and-Mouth Disease Virus in Sheep, Epidemiology and Infection. (1968) 66, no. 4, 633–640, 10.1017/S0022172400028369, 2-s2.0-0014382809. PMC2130666 4303955

[20] McVicar J. W. and Sutmoller P. , Sheep and Goats as Foot-and-Mouth Disease Carriers, 1969, 72, 400–406. 5257477

[21] Hedger R. S. , Foot-and-Mouth Disease and the African Buffalo (*Syncerus caffer*), Journal of Comparative Pathology. (1972) 82, no. 1, 19–28, 10.1016/0021-9975(72)90022-9, 2-s2.0-0015255356. 4336115

[22] Condy J. B. , Hedger R. S. , Hamblin C. , and Barnett I. T. R. , The Duration of the Foot-and-Mouth Disease Virus Carrier State in African Buffalo (i) in the Individual Animal and (ii) in a Free-Living Herd, Comparative-Immunology-Microbiology-and-Infectious-Diseases. (1985) 8, 259–265. 3004803 10.1016/0147-9571(85)90004-9

[23] Alexandersen S. , Zhang Z. , and Donaldson A. I. , Aspects of the Persistence of Foot-and-Mouth Disease Virus in Animals-the Carrier Problem, 2002. 10.1016/s1286-4579(02)01634-9 12191660

[24] Condy J. B. , Hedger R. S. , Hamblin C. , and Barnett I. T. R. , The Duration of the Foot-and-Mouth Disease Virus Carrier State in African Buffalo (i) in the Individual Animal and (ii) in a Free-Living Herd, Comparative Immunology, Microbiology and Infectious Diseases. (1985) 8, no. 3-4, 259–265, 10.1016/0147-9571(85)90004-9, 2-s2.0-0022383052. 3004803

[25] Bengis R. G. , Thomson G. R. , Hedger R. S. , De Vos V. , and Pini A. , Foot-and-Mouth Disease and the African Buffalo (*Syncerus caffer*). 1. Carriers as a Source of Infection for Cattle, The Onderstepoort Journal of Veterinary Research. (1986) 53, no. 2, 69–73. 3014418

[26] Condy J. B. and Hedger R. S. , The Survival of Foot-and-Mouth Disease Virus in African Buffalo With Non-Transference of Infection to Domestic Cattle, Research in Veterinary Science. (1974) 16, 182–185. 4364599

[27] Woodbury E. , A Review of the Possible Mechanisms for the Persistence of Foot-and-Mouth Disease Virus, Epidemiology and Infection. (1995) 114, no. 1, 1–13, 10.1017/S0950268800051864, 2-s2.0-0028925317. 7867727 PMC2271334

[28] Dawe P. S. , Sorensen K. , Sorensen K. , Barnett I. T. , Armstrong R. M. , and Knowles N. J. , Experimental Transmission of Foot-and-Mouth Disease Virus From Carrier African Buffalo (*Syncerus caffer*) to Cattle in Zimbabwe, Veterinary Record. (1994) 134, no. 9, 211–215, 10.1136/vr.134.9.211, 2-s2.0-0028780635. 8171808

[29] Dawe P. S. , Flanagan F. O. , and Madekurozwa R. L. , et al.Natural Transmission of Foot-and-Mouth Disease Virus From African Buffalo (*Syncerus caffer*) to Cattle in a Wildlife Area of Zimbabwe, Veterinary Record. (1994) 134, no. 10, 230–232, 10.1136/vr.134.10.230, 2-s2.0-0028764964. 8197679

[30] Ayebazibwe C. , Tjørnehøj K. , and Mwiine F. N. , et al.Patterns, Risk Factors and Characteristics of Reported and Perceived Foot-and-Mouth Disease (FMD) in Uganda, Tropical Animal Health and Production. (2010) 42, no. 7, 1547–1559, 10.1007/s11250-010-9605-3, 2-s2.0-77956176171. 20526861

[31] Muleme M. , Barigye R. , Khaitsa M. L. , Berry E. , Wamono A. W. , and Ayebazibwe C. , Effectiveness of Vaccines and Vaccination Programs for the Control of Foot-and-Mouth Disease in Uganda, 2001-2010, Tropical Animal Health and Production. (2012) 45, no. 1, 35–43, 10.1007/s11250-012-0254-6, 2-s2.0-85027920810. 22956440

[32] Byamukama B. , Amin A. , Mwiine F. N. , and Ekiri A. B. , Epidemiology and Control Strategies for Foot-and-Mouth Disease in Livestock and Wildlife in Uganda: Systematic Review, Submitted to Transboundary and Emerging Diseases 2024. 10.1007/s11259-025-10791-z PMC12170765 40522510

[33] Munsey A. , Mwiine F. N. , and Ochwo S. , et al.Ecological and Anthropogenic Spatial Gradients Shape Patterns of Dispersal of Foot-and-Mouth Disease Virus in Uganda, Pathogens. (2022) 11, no. 5, 10.3390/pathogens11050524, 524. 35631045 PMC9143568

[34] Velazquez-Salinas L. , Mwiine F. N. , and Ahmed Z. , et al.Genetic Diversity of Circulating Foot and Mouth Disease Virus in Uganda Cross-Sectional Study During 2014-2017, Frontiers in Veterinary Science. (2020) 7, 10.3389/fvets.2020.00162. PMC7109301 32270002

[35] Munsey A. , Mwiine F. N. , and Ochwo S. , et al.Spatial Distribution and Risk Factors for Foot and Mouth Disease Virus in Uganda: Opportunities for Strategic Surveillance, Preventive Veterinary Medicine. (2019) 171, 10.1016/j.prevetmed.2019.104766, 2-s2.0-85072259915, 104766. 31541845

[36] González-Gordon L. , Porphyre T. , and Muwonge A. , et al.Identifying Target Areas for Risk-Based Surveillance and Control of Transboundary Animal Diseases: A Seasonal Analysis of Slaughter and Live-Trade Cattle Movements in Uganda, Scientific Reports. (2023) 13, no. 1, 10.1038/s41598-023-44518-4. PMC10616094 37903814

[37] Okello J. , Okello W. , and Muhanguzi S. , et al.Spatial and Temporal Distribution of Foot and Mouth Disease in Cattle in Uganda from 2010-2021 (A Retrospective Study), Research Square. (2022) 10.21203/rs.3.rs-2013492/v1.

[38] Mwiine F. N. , Velazquez-Salinas L. , and Ahmed Z. , et al.Serological and Phylogenetic Characterization of Foot and Mouth Disease Viruses From Uganda during Cross-Sectional Surveillance Study in Cattle Between 2014 and 2017, Transboundary and Emerging Diseases. (2019) 66, no. 5, 2011–2024, 10.1111/tbed.13249, 2-s2.0-85068098370. 31127983

[39] Kerfua S. D. , Shirima G. , and Kusiluka L. , et al.Spatial and Temporal Distribution of Foot-and-Mouth Disease in Four Districts Situated Along the Uganda-Tanzania Border: Implications for Cross-Border Efforts in Disease Control, Onderstepoort Journal of Veterinary Research. (2018) 85, no. 1, 10.4102/OJVR.V85I1.1528, 2-s2.0-85055071716.

[40] Kerfua S. D. , Dhikusooka M. T. , and Mulondo A. L. , et al.Occurrence of Foot-and-Mouth Disease Virus Serotypes in Uganda and Tanzania (2003 to 2015): A Review and Implications for Prospective Regional Disease Control, Journal of Agricultural Science. (2020) 12, no. 6, 10.5539/jas.v12n6p119, 119.

[41] Baluka S. A. , Economic Effects of Foot and Mouth Disease Outbreaks Along the Cattle Marketing Chain in Uganda, Veterinary World. (2016) 9, no. 6, 544–553, 10.14202/vetworld.2016.544-553, 2-s2.0-84976513296. 27397974 PMC4937042

[42] Statement to Parliament by the Honorable Minister of Agriculture, Animal Husbandry, and Fisheries in Response to Issues Raised by the Right Honorable Speaker of Parliament During the Second Sitting of the Third Meeting of the Thirtieth Session of the Eleventh Parliament on Thursday, Regarding Foot-and-Mouth Disease, 2024, MAAIF FMD situation Update.

[43] Bertram M. R. , Yadav S. , Stenfeldt C. , Delgado A. , and Arzt J. , Extinction Dynamics of the Foot-and-Mouth Disease Virus Carrier State Under Natural Conditions, Frontiers in Veterinary Science. (2020) 7, 10.3389/fvets.2020.00276. PMC7249781 32509810

[44] Jones R. , King D. P. , and Busin V. , Retrospective Analysis of Submissions to the World Reference Laboratory for Foot-and-Mouth Disease: What Can These Data Tell Us About the Role of Small Ruminants in Disease Epidemiology?, Preventive Veterinary Medicine. (2025) 239, 10.1016/j.prevetmed.2025.106526, 106526. 40174344

[45] Balinda S. N. , Tjørnehøj K. , and Muwanika V. B. , et al.Prevalence Estimates of Antibodies Towards Foot-and-Mouth Disease Virus in Small Ruminants in Uganda, Transboundary and Emerging Diseases. (2009) 56, no. 9-10, 362–371, 10.1111/j.1865-1682.2009.01094.x, 2-s2.0-70449449164. 19909475

[46] Namatovu A. , Belsham G. J. , and Ayebazibwe C. , et al.Challenges for Serology-Based Characterization of Foot-and-Mouth Disease Outbreaks in Endemic Areas; Identification of Two Separate Lineages of Serotype O FMDV in Uganda in 2011, Transboundary and Emerging Diseases. (2015) 62, no. 5, 522–534, 10.1111/tbed.12170, 2-s2.0-84940586060. 24118785

[47] Cochran W. G. , Sampling Techniques, 1997, 3rd edition, John Wiley & Sons.

[48] Braun V. and Clarke V. , Cooper H. , Camic P. M. , Long D. L. , Panter A. T. , Rindskopf D. , and Sher K. J. , Thematic Analysis, APA Handbook of Research Methods in Psychology, 2012, 2, American Psychological Association, 57–71, Research Designs: Quantitative, Qualitative, Neuropsychological, and Biological.

[49] Mesfine M. , Nigatu S. , Belayneh N. , and Jemberu W. T. , Sero-Epidemiology of Foot and Mouth Disease in Domestic Ruminants in Amhara Region, Ethiopia, Frontiers in Veterinary Science. (2019) 6, 10.3389/fvets.2019.00130, 2-s2.0-85064322761. PMC6503644 31114792

[50] Chepkwony E. C. , Gitao G. C. , Muchemi G. M. , Sangula A. K. , and Kairu-Wanyoike S. W. , Epidemiological Study on Foot-and-Mouth Disease in Small Ruminants: Sero-Prevalence and Risk Factor Assessment in Kenya, PLoS ONE. (2021) 16, no. 8, 10.1371/journal.pone.0234286, e0234286. 34339447 PMC8328338

[51] Raouf Y. A. , Yousif H. , Almutlab A. A. , Hassen A. A. , Al-Majali A. , and Tibbo M. , Role of Small Ruminants in the Epidemiology of Foot-and-Mouth Disease in Sudan, Bulletin of Animal Health and Production in Africa. (2018) 65, 145–156.

[52] Di N. A. , Ferretti L. , and Wadsworth J. , et al.Evolutionary and Ecological Drivers Shape the Emergence and Extinction of Foot-and-Mouth Disease Virus Lineages, Molecular Biology and Evolution. (2021) 38, no. 10, 4346–4361, 10.1093/molbev/msab172. 34115138 PMC8476141

[53] Hughes G. J. , Kitching R. P. , and Woolhouse M. E. J. , Dose-Dependent Responses of Sheep Inoculated Intranasally With a Type O Foot-and-Mouth Disease Virus, Journal of Comparative Pathology. (2002) 127, no. 1, 22–29, 10.1053/jcpa.2002.0560, 2-s2.0-0036620679. 12354542

[54] Gibson C. F. , Donaldson A. I. , and Ferris N. P. , Response of Sheep Vaccinated With Large Doses of Vaccine to Challenge by Airborne Foot and Mouth Disease Virus, Vaccine. (1984) 2, no. 2, 157–161, 10.1016/0264-410X(84)90008-2, 2-s2.0-0021178087. 6099647

[55] Alice N. , Tjørneh K. , and Belsham G. J. , et al.Characterization of Foot-And-Mouth Disease Viruses (FMDVs) From Ugandan Cattle Outbreaks During 2012-2013: Evidence for Circulation of Multiple Serotypes, PLoS ONE. (2015) 10, no. 2, 10.1371/journal.pone.0114811, 2-s2.0-84922600025, e0114811. 25664876 PMC4321839

[56] Mielke S. R. and Garabed R. , Environmental Persistence of Foot-and-Mouth Disease Virus Applied to Endemic Regions, Transboundary and Emerging Diseases. (2020) 67, no. 2, 543–554, 10.1111/tbed.13383. 31595659

[57] Mielke S. R. , Lendzele S. , Delgado A. H. , Abdoulmoumini M. , Dickmu S. , and Garabed R. , Patterns of Foot-and-Mouth Disease Virus Detection in Environmental Samples in an Endemic Setting, Frontiers in Veterinary Science. (2023) 10, 10.3389/fvets.2023.1157538. PMC10312077 37396995

[58] Vosloo W. , Boshoff K. , Dwarka R. , and Bastos A. , The Possible Role That Buffalo Played in the Recent Outbreaks of Foot-and-Mouth Disease in South Africa, Annals of the New York Academy of Sciences. (2002) 969, 187–190, 10.1111/j.1749-6632.2002.tb04376.x, 2-s2.0-0036411420. 12381589

[59] Thomson G. R. , Vosloo W. , and Bastos A. D. S. , Foot and Mouth Disease in Wildlife, 2003. 10.1016/s0168-1702(02)00263-0 12527441

[60] Casey-Bryars M. , The Epidemiology of Foot-and-Mouth Disease at the Wildlife-Livestock Interface in Northern Tanzania, 2016, University of Glasgow.

[61] Bastos A. D. S. , Haydon D. T. , Sangaré O. , Boshoff C. I. , Edrich J. L. , and Thomson G. R. , The Implications of Virus Diversity Within the SAT. 2 Serotype for Control of Foot-and-Mouth Disease in Sub-Saharan Africa, Journal of General Virology. (2003) 84, no. 6, 1595–1606, 10.1099/vir.0.18859-0, 2-s2.0-0037639110. 12771430

[62] Bastos A. D. S. , Anderson E. C. , Bengis R. G. , Keet D. F. , Winterbach H. K. , and Thomson G. R. , Molecular Epidemiology of SAT3-Type Foot-and-Mouth Disease, Virus Genes. (2003) 27. 10.1023/a:1026352000959 14618089

[63] UBOS , National Livestock Census 2021 Abridged Version Uganda Bureau Of Statistics The Republic Of Uganda, 2024.

[64] Ahmed B. , Megersa L. , Mulatu G. , Siraj M. , and Boneya G. , Seroprevalence and Associated Risk Factors of Foot and Mouth Disease in Cattle in West Shewa Zone, Ethiopia, Veterinary Medicine International. (2020) 2020, 10.1155/2020/6821809, 6821809. 32292580 PMC7150713

[65] Sulayeman M. , Dawo F. , Mammo B. , Gizaw D. , and Shegu D. , Isolation, Molecular Characterization and Sero-Prevalence Study of Foot-and-Mouth Disease Virus Circulating in Central Ethiopia, BMC Veterinary Research. (2018) 14, no. 1, 10.1186/s12917-018-1429-9, 2-s2.0-85044404869. PMC5870258 29587741

[66] Sumption K. , Domenech J. , and Ferrari G. , Progressive Control of FMD on a Global Scale, Veterinary Record. (2012) 170, no. 25, 637–639, 10.1136/vr.e4180, 2-s2.0-84862874029. 22730497

[67] FAO and OIE , The Global Foot and Mouth Disease Control Strategy: Strengthening Animal Health Systems Through Improved Control of Major Diseases, 2012.

[68] Assefa S. , Duguma B. , and Worku Z. , Assessment of Livestock Husbandry Practices and Production Constraints Among Smallholder Mixed Crop-Livestock Production Systems in the Majang Zone, Southwest Ethiopia, Heliyon. (2024) 10, no. 19, 10.1016/j.heliyon.2024.e37400, e37400. 39381094 PMC11456831

