# Supplementary material for: Epidemiology of Foot‐and‐Mouth Disease in Goats in Uganda: A Risk‐Based Approach
Source: Transbound Emerg Dis. 2026 Apr 15;2026:2808139. doi: 10.1155/tbed/2808139 (PMC13080341; doi:10.1155/tbed/2808139)
Supplement: Supplementary file 1 — Supporting Information 1 File S1: Questionnaire used to collect data on farm management, FMD history, disease control practices, and other related information. [file TBED-2026-2808139-s001.docx]

Supplementary File 1

Questionnaire

Start of Block: PART 1: Farm Information

**In which district is your farm located?**

________________________________________________________________

**In which subcounty is your farm located?**

________________________________________________________________

**In which parish is your farm located?**

________________________________________________________________

**In which village is your farm located?**

________________________________________________________________

**What is the farm name or owner's name?**

________________________________________________________________

**For how long you have been keeping livestock (cattle, sheep, goats, pigs)?**

- 5 to 10 years (4)
- 10 to 15 years (5)
- 15 to 20 years (6)
- More than 20 years (7)

**Which type of livestock do you keep at your farm?**

- Cattle (1)
- Sheep or goats (2)
- Pigs (3)

Display This Question:

If Which type of livestock do you keep at your farm? = Cattle

**If you keep cattle, how many cattle and breeds do you keep?**

- Local breed (Ankole, Nganda, East African short-horn) (4) __________________________________________________
- Cross breed (5) __________________________________________________
- Pure exotic (6) __________________________________________________

Display This Question:

If Which type of livestock do you keep at your farm? = Cattle

**If you keep cattle, which type of farming do you practice (select all that apply)**

- Pastoral system (1)
- Agro-pastoral system (2)
- Ranching (3)
- Paddock (4)
- Zero-grazing (5)
- Free-range (6)
- Other please specify (7) __________________________________________________

Display This Question:

If Which type of livestock do you keep at your farm? = Sheep or goats

**If you keep sheep or goats, how many do you keep?**

- Sheep (4) __________________________________________________
- Goat (5) __________________________________________________

Display This Question:

If Which type of livestock do you keep at your farm? = Sheep or goats

**If you keep sheep or goats, what farming system do you use?**

- Extensive / traditional system (Mainly managed under free-range, graze on communal or unimproved natural pastures with minimal inputs are provided, and often graze in mixed herds) (1)
- Semi-Intensive system including Tethering (Kept in paddocks, tether or fenced enclosures with occasional grazing and farmers may supplement feeding with crop residues and purchased feed) (2)
- Intensive system (Animals kept in confined areas and provided with all their feed, water, and veterinary care). (3)
- Other (please specify) (4) __________________________________________________

Display This Question:

If Which type of livestock do you keep at your farm? = Pigs

**If you keep pigs, how many do you keep?**

________________________________________________________________

Display This Question:

If Which type of livestock do you keep at your farm? = Pigs

**If you keep pigs, which farming system do you utilize?**

- Extensive/ scavenging system (Pigs left to roam freely around villages and scavenge for food, feeding on household waste, crop residues, and forages). (1)
- Semi-intensive system including Tethering (Pigs kept in enclosures, tether, or paddocks with access to external grazing and farmers provide supplemental feeds, such as crop residues, kitchen waste, or commercial feed). (2)
- Intensive/ commercial system (Pigs are confined and provided with all their feed, water, and veterinary care. (3)
- Other (please specify) (4) __________________________________________________

End of Block: PART 1: Farm Information

Start of Block: PART 2: FMD Outbreak History

**When was the last time you observed an FMD case on your farm?**

- Less than 3 months ago (6)
- Less than 6 months ago (1)
- 6-12 months ago (2)
- Greater 1 year to 2 years ago (3)
- Over 2 years ago (4)
- Never (5)
- Others (please specify) (7) __________________________________________________

**Thinking of the last event when you had an FMD case on your farm, did you observe clinical signs in the affected animals?**

- Yes (1)
- No (2)
- Do not know or do not remember (4)

Display This Question:

If Thinking of the last event when you had an FMD case on your farm, did you observe clinical signs... = Yes

**If you observed clinical signs in affected animals, in what animal species did you observe the signs? Select all that apply.**

- Cattle (1)
- Sheep (2)
- Goat (3)
- Pig (4)
- Other (please specify) (5) __________________________________________________

Display This Question:

If Thinking of the last event when you had an FMD case on your farm, did you observe clinical signs... = Yes

**If you observed clinical signs in affected animals, what signs did you observe (select all that apply)?**

- Fever (1)
- Lameness (2)
- Loss of appetite (3)
- Blisters in mouth or foot (4)
- Wound in the mouth (5)
- Salivation (7)
- Other (Please specify) (6) __________________________________________________

**Have your neighbours within your village or parish or subcounty reported FMD cases in the past?**

- Yes (1)
- No (2)
- I do not know or not sure (3)

Display This Question:

If Have your neighbours within your village or parish or subcounty reported FMD cases in the past? = Yes

**If yes, when did your neighbours within your village or parish or subcounty report FMD cases?**

- Less than 3 months ago (5)
- Less than 6 months ago (1)
- 6-12 months ago (2)
- Greater than 1 to 2 years ago (3)
- Over 2 years ago (4)
- Do not know (6)

**What is the approximate distance to the nearest farm/household with livestock (cattle, sheep, goats, pigs)?**

- Less than 1 km (1)
- 1-5 km (2)
- 6-10 km (3)
- Over 10 km (4)
- Do not know (5)

**Do you purchase animals from livestock markets or from fellow farmers? Select all that apply.**

- Yes I buy from livestock markets (1)
- Yes I buy from fellow farmers (3)
- No (4)
- Others (please specify) (5) __________________________________________________

**If you purchase animals from livestock markets or from fellow farmers, how many animals did you purchase in the last 12 months?**

________________________________________________________________

Display This Question:

If Which type of livestock do you keep at your farm? = Cattle

**Do you make hay or silage for cattle on your farm?**

- Yes (1)
- No (2)

Display This Question:

If Which type of livestock do you keep at your farm? = Sheep or goats

**Do you make hay or silage for sheep or goats on your farm?**

- Yes (1)
- No (2)

Display This Question:

If Which type of livestock do you keep at your farm? = Pigs

**Do you feed your pigs on feed supplements (for example kitchen leftovers)?**

- Yes (1)
- No (2)

**Have you observed any interactions between your livestock (cattle, sheep, goats, or pigs) and wild animals (such as buffalo, warthogs, deer, antelope) in the past 1-2 years?**

- Yes (1)
- No (2)

Display This Question:

If Have you observed any interactions between your livestock (cattle, sheep, goats, or pigs) and wil... = Yes

**If yes, what type of wildlife do your cattle interact with? (Select all that apply).**

- Buffaloes (1)
- Warthogs (2)
- Antelopes (3)
- Zebras (4)
- Elephants (5)
- Others (please specify) (6) __________________________________________________

Display This Question:

If Have you observed any interactions between your livestock (cattle, sheep, goats, or pigs) and wil... = Yes

**If yes, what type of wildlife do your sheep or goats interact with? (Select all that apply).**

- Buffaloes (1)
- Warthogs (2)
- Antelopes (3)
- Zebras (4)
- Elephants (5)
- Others (please specify) (6) __________________________________________________

Display This Question:

If Have you observed any interactions between your livestock (cattle, sheep, goats, or pigs) and wil... = Yes

**If yes, what type of wildlife do your pigs interact with? (Select all that apply).**

- Buffaloes (1)
- Warthogs (2)
- Antelopes (3)
- Zebras (4)
- Elephants (5)
- Others (please specify) (6) __________________________________________________

End of Block: PART 2: FMD Outbreak History

Start of Block: Animal movements

**Do you move your animals (cattle or sheep of goats) outside your farm in search of pasture or water?**

- Yes (1)
- No (2)

Display This Question:

If Do you move your animals (cattle or sheep of goats) outside your farm in search of pasture or water? = Yes

**If you move your animals outside your farm in search of pasture or water, how often do you move your animals?**

- Daily (1)
- Weekly (2)
- Monthly (3)
- Seasonally especially during dry season (4)
- Never (5)
- Others (please specify) (6) __________________________________________________

**Have you moved your cattle, sheep, or goats across district or national borders in the past year?**

- Yes (1)
- No (2)

**Do you move your animals (cattle or sheep or goats) outside your farm due to other reasons? If yes, describe the reasons below.**

________________________________________________________________

End of Block: Animal movements

Start of Block: Communal grazinng

**Do you share grazing areas for your cattle or sheep or goats with other farmers?**

- Yes (1)
- No (2)

Display This Question:

If Do you share grazing areas for your cattle or sheep or goats with other farmers? = Yes

**How often do your cattle or sheep or goats graze communally with those of other farmers?**

- Daily (1)
- Weekly (2)
- Monthly (3)
- Seasonally (4)
- Others (please specify) (5) __________________________________________________

End of Block: Communal grazinng

Start of Block: Seasonal Factors

**During the dry season, do you move your animals to communal or shared grazing and watering areas?**

- Yes (1)
- No (2)

Display This Question:

If During the dry season, do you move your animals to communal or shared grazing and watering areas? = Yes

**If yes, how far do you typically move your animals?**

- Less than 5 km (1)
- 5-10 km (2)
- More than 10 km (3)
- Others (Please specify) (4) __________________________________________________

End of Block: Seasonal Factors

Start of Block: Proximity Factors

**Is your farm located near an international border?**

- Yes (1)
- No (2)

Display This Question:

If Is your farm located near an international border? = Yes

**If yes, how far is your farm from the border?**

- Less than 5 km (1)
- 5-10 km (2)
- More than 10 km (3)
- Do not know (5)

**Is your farm located near a national park?**

- Yes (1)
- No (2)

Display This Question:

If Is your farm located near a national park? = Yes

**If yes, how far is your farm from the nearest national park?**

- Less than 5 km (1)
- 5-10 km (2)
- More than 10 km (3)
- Do not know (5)

End of Block: Proximity Factors

Start of Block: PART 3: Biosecurity Measures

**Is your farm (where animals are kept) fully fenced or enclosed by a fence?**

- Yes (1)
- No (2)

**Is fence or enclosure able to prevent entry or exit of animals from your farm/household?**

- Yes (4)
- No (5)

**Is there controlled access at your farm (gate or other)?**

- Yes (1)
- No (2)

**Is there footbath/tyre bath (area for disinfecting shoes/boots/vehicle wheels) at the entry to your farm?**

- Yes (1)
- No (2)

**If there is a footbath/tyre bath (area for disinfecting shoes/boots/vehicle wheels), is it currently in use?**

- Yes (4)
- No (5)

**Which of the following measures do you practice on your farm to prevent the spread of FMD in animals? (Select all that apply)**

- Isolation of new animals (1)
- Regular veterinary check-ups (2)
- Vaccination (3)
- Other (Please specify) (4) __________________________________________________

Display This Question:

If Which of the following measures do you practice on your farm to prevent the spread of FMD in anim... = Vaccination

**When did you conduct the last FMD vaccination of cattle at your farm?**

- Less than 6 months ago (1)
- 1 year ago (2)
- 1.5 years ago (3)
- 2 years ago (4)
- More than 3 years ago (5)
- Never (6)
- I don't know (7)

Display This Question:

If Which of the following measures do you practice on your farm to prevent the spread of FMD in anim... = Vaccination

**What is the frequency of FMD vaccination of cattle at your farm?**

- Twice a year (1)
- Once a year (2)
- Once in 2 years (3)
- Only during outbreaks (4)
- Others (please specify) (5) __________________________________________________

Display This Question:

If Which of the following measures do you practice on your farm to prevent the spread of FMD in anim... = Vaccination

**If you have vaccinated against FMD, what was the source of the vaccine?**

- Government (1)
- Private vets (2)
- FAO (4)
- Others (please specify) (3) __________________________________________________

End of Block: PART 3: Biosecurity Measures

Start of Block: Additional Information

**Have you received any training or information on FMD prevention and control in the last two years?**

- Yes (1)
- No (2)

Display This Question:

If Have you received any training or information on FMD prevention and control in the last two years? = Yes

**If you have training on FMD prevention and control, how often do you receive the training?**

- Twice a year (5)
- Three times a year (6)
- Other (please specify) (4) __________________________________________________

Display This Question:

If Have you received any training or information on FMD prevention and control in the last two years? = Yes

**If yes, from whom did you receive the training? List below.**

________________________________________________________________

**In your opinion, what are the key challenges you face on your farm or in the community to prevent the spread of FMD in animals?**

________________________________________________________________

End of Block: Additional Information
