# Supplementary material for: Epidemiology of Foot‐and‐Mouth Disease in Goats in Uganda: A Risk‐Based Approach
Source: Transbound Emerg Dis. 2026 Apr 15;2026:2808139. doi: 10.1155/tbed/2808139 (PMC13080341; doi:10.1155/tbed/2808139)
Supplement: Supplementary file 2 — Supporting Information 2 Table S1: The selection criteria of the study districts following a risk‐based approach. Supporting Table S2: Animal level, farm level, and district level seroprevalence. Supporting Table S3: Results of univariable logistic regression—factors associated with FMD seropositivity. Supporting Table S4: Results of multicollinearity assessment. Supporting Table S5: Results of multivariable logistic regression analyses—seven different models evaluated. [file TBED-2026-2808139-s002.docx]

**SUPPLEMENTARY TABLES**

**Supplementary Table 1**: Showing the selection criteria of the study districts following a risk-based approach.

**Supplementary Table 2**: Animal level, farm level and district level seroprevalence.

**Supplementary Table 3**: Results of univariable logistic regression - factors associated with FMD seropositivity.

**Supplementary Table 4**: Results of multicollinearity assessment.

**Supplementary Table 5**: Results of multivariable logistic regression analyses - seven different models evaluated.

**Supplementary Table 1**: Showing the selection criteria of the study districts following a risk-based approach.

| **Districts with recent FMD outbreaks (Feb 2024)** | **Region** | **Subregion** | **Period of FMD outbreaks (month & year)** | **Latest cases**  **(month & year)** | **Border district** | **Wildlife livestock interface** | **Drought prone district** | **Communal grazing** | **High livestock density** |
| --- | --- | --- | --- | --- | --- | --- | --- | --- | --- |
| Budaka | Eastern | Eastern | Nov 2023 |  |  |  |  |  |  |
| Bukedea | Eastern | Teso | Dec 2023 |  |  |  |  |  |  |
| Bukomansimbi | Central | Central | Dec 2023 |  |  |  |  |  |  |
| Bunyangabu | Western | Western | Dec 2023 |  |  | Yes (Kibaale NP) |  |  |  |
| Butaleja | Eastern | Eastern | Dec 2023 |  |  |  |  |  |  |
| Fortportal City | Western | Western | Dec 2023 | Apr 2024 |  |  |  |  |  |
| Gomba | Central | Central | Feb 2023 | Mar 2024 |  |  |  |  | Yes |
| Ibanda | Western | Western | Dec 2023 |  |  |  |  |  | Yes |
| Isingiro | Western | Southwestern | Dec 2023 |  | Yes | Yes (LMNP) | Yes | Yes | Yes |
| Kabarole | Western | Western | Feb 2024 | Apr 2024 |  | Yes (Kibaale NP and Semliki NP) |  |  |  |
| Kasese | Western | Rwenzori | Jan 2024 |  | Yes | Yes (QENP) |  | Yes |  |
| Kassanda | Central | Central | Dec 2024 |  |  |  |  |  | Yes |
| Kayunga | Central | Central | Dec 2024 |  |  |  |  |  | Yes |
| Kazo | Central | Central | Dec 2024 | Jun 2024 |  | Yes (LMNP) |  |  | Yes |
| Kiboga | Central | Central | Dec 2024 | May2024 |  |  | Yes | Yes | Yes |
| Kibuku | Eastern | Eastern | Jan 2024 | Sep 2024 |  |  |  |  |  |
| Kiruhura | Western | Southwestern | Dec 2024 |  |  | Yes (LMNP) | Yes | Yes | Yes |
| Kumi | Eastern | Teso | Dec 2024 |  |  |  |  |  | Yes |
| Kyankwanzi | Central | Central | Dec 2024 | Jan 2024 |  |  | Yes |  | Yes |
| Kyegegwa | Western | Western | Dec 2024 | Jan 2024 |  |  |  | Yes |  |
| Kyotera | Central | Central | Dec 2024 |  | Yes |  |  |  | Yes |
| Luuka | Eastern | Eastern | Dec 2024 |  |  |  |  |  |  |
| Lwengo | Central | Central | Dec 2024 |  |  |  |  |  | Yes |
| Lyantonde | Western | Southwestern | Dec 2024 | Feb 2024 |  | Yes (LMNP) | Yes | Yes | Yes |
| Mbarara | Western | Western | Dec 2024 | Jan 2024 |  |  |  |  | Yes |
| Mbarara City | Western | Western | Dec 2024 | Jan 2024 |  |  |  |  | Yes |
| Mityana | Central | Central | Dec 2024 |  |  |  |  |  |  |
| Mpigi | Central | Central | Dec 2024 |  |  |  |  |  |  |
| Mubende | Central | Central | Feb 2024 | Jun 2024 |  |  |  |  | Yes |
| Nakaseke | Central | Central | Dec 2024 |  |  |  | Yes |  | Yes |
| Nakasongola | Central | Central | Feb 2024 |  |  |  | Yes | Yes | Yes |
| Naminsindwa | Eastern | Elgon | Dec 2024 |  | Yes |  |  |  |  |
| Ngora | Eastern | Teso | Dec 2024 |  |  |  | Yes |  | Yes |
| Ntungamo | Western | Southwestern | Feb 2024 | Mar 2024 | Yes |  |  |  | Yes |
| Rakai | Central | Central | Dec 2024 |  | Yes | Yes (LMNP) | Yes |  | Yes |
| Rwampara | Western | Western | Dec 2024 |  |  |  |  | Yes |  |
| Sembabule. | Central | Central | Feb 2024 | Apr 2024 |  | Yes (LMNP) | Yes | Yes | Yes |
| Sheema | Western | Western | Feb 2024 | Mar 2024 |  | Yes (LMNP) |  |  |  |
| Ntoroko | Western | Western | Jan 2024 | Jun 2024 | Yes | Yes (Semliki NP) |  |  | Yes |

Kibaale NP = Kibaale National Park, LMNP = Lake Mburo National Park, Semliki NP = Semliki National Park, QENP = Queen Elizabeth national Park.

**Supplementary Table 2**: Animal level, farm level and district level seroprevalence.

| **District** | **Subcounty** | **Unique Farm code** | **Number of goats tested** | **Positive** | **Negative** | **%positive per farm** | **95% CI (Lower)** | **95% CI (Upper)** |
| --- | --- | --- | --- | --- | --- | --- | --- | --- |
| Kasese | Muhokya TC | km_muh_v | 10 | 0 | 10 | 0.0 |  |  |
|  |  | bj_muh_v | 10 | 0 | 10 | 0.0 |  |  |
|  |  | ks_muh_v | 10 | 0 | 10 | 0.0 |  |  |
|  |  | sb_muh_v | 10 | 2 | 8 | 20.0 |  |  |
|  |  | ka_muh_v | 10 | 1 | 9 | 10.0 |  |  |
|  |  | mk__muh_v | 10 | 0 | 10 | 0.0 |  |  |
|  |  | vk_muh_v | 10 | 0 | 10 | 0.0 |  |  |
|  |  | mb_muh_v | 10 | 0 | 10 | 0.0 |  |  |
|  | Kitswamba | md_kit_v | 10 | 4 | 6 | 40.0 |  |  |
|  |  | np_kit_v | 12 | 2 | 10 | 16.7 |  |  |
|  |  | sr_kit_v | 10 | 0 | 10 | 0.0 |  |  |
|  |  | ma_kit_v | 10 | 1 | 9 | 10.0 |  |  |
|  |  | bj_kit_v | 13 | 3 | 10 | 23.1 |  |  |
|  |  | bs_kit_v | 10 | 0 | 10 | 0.0 |  |  |
|  |  | kh_kit_v | 10 | 4 | 6 | 40.0 |  |  |
|  |  | ke_kit_v | 13 | 3 | 10 | 23.1 |  |  |
|  |  | **Total** | **160** | **20** | **140** | **12.5** | **8.24** | **18.52** |
| Kiboga | Kapeke | mf2_kap_y | 15 | 0 | 15 | 0.0 |  |  |
|  |  | mf_kap_y | 15 | 3 | 12 | 20.0 |  |  |
|  |  | nj_kap_y | 15 | 1 | 14 | 6.7 |  |  |
|  |  | mr_kap_y | 10 | 2 | 8 | 20.0 |  |  |
|  |  | mc_kap_y | 10 | 0 | 10 | 0.0 |  |  |
|  |  | kj_kap_y | 10 | 2 | 8 | 20.0 |  |  |
|  |  | ap_kap_y | 10 | 1 | 9 | 10.0 |  |  |
|  |  | tjb_kap_y | 15 | 0 | 15 | 0.0 |  |  |
|  |  | kr_kap_y | 15 | 7 | 8 | 46.7 |  |  |
|  | Kayera | tj_kay_y | 10 | 3 | 7 | 30.0 |  |  |
|  |  | tb_kay_y | 15 | 1 | 14 | 6.7 |  |  |
|  |  | bj_kay_y | 15 | 2 | 13 | 13.3 |  |  |
|  |  | **Total** | **155** | **22** | **133** | **14.2** | **9.56** | **20.56** |
| Kiruhura | Akayanja | ks_aka_x | 10 | 0 | 10 | 0.0 |  |  |
|  |  | kf_aka_x | 10 | 4 | 6 | 40.0 |  |  |
|  |  | mg_aka_x | 10 | 7 | 3 | 70.0 |  |  |
|  |  | em_aka_x | 10 | 7 | 3 | 70.0 |  |  |
|  |  | kb_aka_x | 9 | 5 | 4 | 55.6 |  |  |
|  |  | ne_aka_x | 10 | 6 | 4 | 60.0 |  |  |
|  |  | kj_aka_x | 10 | 1 | 9 | 10.0 |  |  |
|  |  | td_aka_x | 10 | 8 | 2 | 80.0 |  |  |
|  |  | ke_aka_x | 10 | 2 | 8 | 20.0 |  |  |
|  | Nyakashashara | tp_nya_x | 10 | 5 | 5 | 50.0 |  |  |
|  |  | gt_nya_x | 10 | 3 | 7 | 30.0 |  |  |
|  |  | sy_nya_x | 10 | 0 | 10 | 0.0 |  |  |
|  |  | pm_nya_x | 10 | 0 | 10 | 0.0 |  |  |
|  |  | kf_nya_x | 10 | 3 | 7 | 30.0 |  |  |
|  |  | eb_nya_x | 10 | 4 | 6 | 40.0 |  |  |
|  |  | sw_nya_x | 10 | 2 | 8 | 20.0 |  |  |
|  |  | mf_nya_x | 10 | 1 | 9 | 10.0 |  |  |
|  |  | tm_nya_x | 10 | 0 | 10 | 0.0 |  |  |
|  |  | **Total** | **179** | **58** | **121** | **32.4** | **25.98** | **39.57** |
| Nakasongola | Nakitoma | tj_nak-w | 10 | 3 | 7 | 30.0 |  |  |
|  |  | ly_nak-w | 10 | 5 | 5 | 50.0 |  |  |
|  |  | ks_nak-w | 10 | 0 | 10 | 0.0 |  |  |
|  |  | bd_nak-w | 10 | 8 | 2 | 80.0 |  |  |
|  |  | ky_nak-w | 10 | 0 | 10 | 0.0 |  |  |
|  |  | bi_nak-w | 10 | 2 | 8 | 20.0 |  |  |
|  |  | mg_nak-w | 10 | 5 | 5 | 50.0 |  |  |
|  |  | ki_nak-w | 10 | 0 | 10 | 0.0 |  |  |
|  | Nabiswera | kas_nab_w | 10 | 0 | 10 | 0.0 |  |  |
|  |  | knb_nab_w | 10 | 1 | 9 | 10.0 |  |  |
|  |  | kem_nab_w | 9 | 3 | 6 | 33.3 |  |  |
|  |  | mum_nab_w | 10 | 2 | 8 | 20.0 |  |  |
|  |  | lui_nab_w | 10 | 0 | 10 | 0.0 |  |  |
|  |  | bys_nab_w | 10 | 0 | 10 | 0.0 |  |  |
|  |  | mus_nab_w | 10 | 0 | 10 | 0.0 |  |  |
|  |  | nas_nab_w | 10 | 0 | 10 | 0.0 |  |  |
|  |  | **Total** | **159** | **30** | **129** | **18.9** | **13.55** | **25.66** |
| Rakai | Greater Kibanda | sc_kib_z | 10 | 5 | 5 | 50.0 |  |  |
|  |  | ke2__kib_z | 9 | 4 | 5 | 44.4 |  |  |
|  |  | kr_kib_z | 10 | 0 | 10 | 0.0 |  |  |
|  |  | ke_kib_z | 10 | 3 | 7 | 30.0 |  |  |
|  |  | snk_kib_z | 10 | 2 | 8 | 20.0 |  |  |
|  |  | tj_kib_z | 11 | 1 | 10 | 9.1 |  |  |
|  |  | kj_kib_z | 10 | 0 | 10 | 0.0 |  |  |
|  |  | dtc_kib_z | 11 | 3 | 8 | 27.3 |  |  |
|  |  | bg_kib_z | 10 | 0 | 10 | 0.0 |  |  |
|  |  | nr_kib_z | 10 | 3 | 7 | 30.0 |  |  |
|  | Kacheera | yw_Kac_z | 10 | 0 | 10 | 0.0 |  |  |
|  |  | bs_Kac_z | 10 | 0 | 10 | 0.0 |  |  |
|  |  | mc_Kac_z | 10 | 10 | 0 | 100.0 |  |  |
|  |  | mj_Kac_z | 10 | 1 | 9 | 10.0 |  |  |
|  |  | km_Kac_z | 10 | 4 | 6 | 40.0 |  |  |
|  |  | kj_kac_z | 10 | 0 | 10 | 0.0 |  |  |
|  |  | gk_kac_z | 10 | 0 | 10 | 0.0 |  |  |
|  |  | ym_kac_z | 10 | 0 | 10 | 0.0 |  |  |
| **Total** |  |  | **179** | **35** | **144** | **19.6** | **14.41** | **25.98** |
| **Overall seropositivity** | | | **832** | **165** | **667** | **19.8** | **17.26** | **22.68** |

**Supplementary Table 3**: Results of univariable logistic regression - factors associated with FMD seropositivity.

| **Variable** | **Categories** | **FMD status** | | | **Estimates** | | | |
| --- | --- | --- | --- | --- | --- | --- | --- | --- |
|  |  | **Negative** | **Positive** | **Total** | **OR** | **95% C.I.** | | ***p*-value** |
|  |  | **n (%)** | **n (%)** | **n (%)** |  | **Lower** | **Upper** |  |
| **1. Animal-level factors associated with FMD seropositivity** | | | | | | | | |
| **Breed** | Cross breed | 257 (46.1%) | 54 (36.0%) | 311 (44.0%) | 0.657 | 0.452 | 0.953 | 0.027 |
|  | Local breeds | 300 (53.9%) | 96 (64.0%) | 396 (56.0%) | REF |  |  |  |
| **Age** | > 1 year | 585 (87.7%) | 150 (90.9%) | 735 (88.3%) |  |  |  |  |
|  | ≤ 1 year | 82 (12.3%) | 15 (9.1%) | 97 (11.7%) | 0.713 | 0.400 | 1.273 | 0.253 |
| **Sex** | Female | 579 (86.8%) | 149 (90.3%) | 728 (87.5%) | 1.415 | 0.807 | 2.484 | 0.226 |
|  | Male | 88 (13.2%) | 16 (9.7%) | 104 (12.5%) | REF |  |  |  |
| **Body condition score** | >2.5 | 305 (93.0%) | 100 (92.6%) | 405 (92.9%) | REF |  |  |  |
|  | ≤2.5 | 23 (7.0%) | 8 (7.4%) | 31 (7.1%) | 1.061 | 0.460 | 2.447 | 0.890 |
| **2. Farm-level factors related to FMD occurrence and history** | | | | | | | | |
| **District of origin** | Kasese | 140 (21.0%) | 20 (12.1%) | 1 60 (19.2%) | 0.864 | 0.451 | 1.655 | 0.659 |
|  | Kiboga | 133 (19.9%) | 22 (13.3%) | 155 (18.6%) | REF |  |  |  |
|  | Kiruhura | 121 (18.1%) | 58 (35.2%) | 179 (21.5%) | 2.898 | 1.673 | 5.018 | <0.001 |
|  | Nakasongola | 129 (19.3%) | 30 (18.2%) | 159 (19.1%) | 1.406 | 0.771 | 2.565 | 0.267 |
|  | Rakai | 144 (21.6%) | 35 (21.2%) | 179 (21.5%) | 1.469 | 0.820 | 2.632 | 0.196 |
| **FMD history at the farm** | <6 months ago, | 186 (22.4%) | 68 (41.2%) | 254 (30.5%) | 3.224 | 2.046 | 5.08 | <.001 |
|  | >6-12 months ago | 53 (6.4%) | 17 (10.3%) | 70 (8.4%) | 2.828 | 1.47 | 5.441 | 0.002 |
|  | >1 year ago, | 137 (16.5%) | 47 (28.5%) | 184 (22.1%) | 3.025 | 1.855 | 4.934 | <.001 |
|  | Never | 291 (30.5%) | 33 (20.0%) | 324 (38.9%) | REF |  |  |  |
| **Last FMD case on farm** | Less than 3 months ago | 53 (8.0%) | 32  (19.6%) | 85 (10.3%) | 4.830 | 2.762 | 8.448 | <0.001 |
|  | Less than 6 months ago | 133 (20.2%) | 36 (22.1%) | 169 (20.6%) | 2.165 | 1.306 | 3.590 | 0.003 |
|  | >6-12 months ago | 53 (8.0%) | 17 (10.4%) | 70 (8.5%) | 2.566 | 1.344 | 4.900 | 0.004 |
|  | >1 year to 2 years ago | 62 (9.4%) | 17 (10.4%) | 79 (9.6%) | 2.194 | 1.158 | 4.155 | 0.016 |
|  | >2 years ago, | 70 (10.6%) | 25 (15.3%) | 95 (11.6%) | 2.857 | 1.611 | 5.068 | <0.001 |
|  | Never | 288 (43.7%) | 36 (22.1%) | 324 (39.4%) | REF |  |  |  |
| **Observed any clinical signs** | Yes | 357 (67.4%) | 126 (91.3%) | 483 (72.3%) | 3.497 | 1.821 | 6.716 | <0.001 |
|  | No | 109 (20.6%) | 11 (8.0%) | 120 (18.0%) | REF |  |  |  |
|  | Do not know or do not remember | 64 (12.1%) | 1 (0.7%) | 65 (9.7%) | 0.155 | 0.020 | 1.227 | 0.077 |
| **Neighbours had FMD** | Yes | 488 (76.4%) | 149 (91.4%) | 637 (79.4%) | 2.396 | 1.307 | 4.390 | 0.005 |
|  | No | 102 (16.0%) | 13 (8.0%) | 115 (14.3%) | REF |  |  |  |
|  | I do not know or not sure | 49 (7.7%) | 1 (0.6%) | 50 (6.2%) | 0.160 | 0.020 | 1.259 | 0.082 |
| **Period when neighbours had FMD** | Less than 3 months ago | 60 (12.3%) | 39 (26.2%) | 99 (15.5%) | 3.427 | 1.602 | 7.330 | 0.001 |
|  | Less than 6 months ago | 239 (49.0%) | 70 (47.0%) | 309 (48.5%) | 1.544 | 0.769 | 3.102 | 0.222 |
|  | 6-12 months ago | 131 (26.8%) | 29 (19.5%) | 160 (25.1%) | 1.167 | 0.546 | 2.495 | 0.690 |
|  | Greater than 1 to 2 years ago (ref) | 58 (11.9%) | 11 (7.4%) | 69 (10.8%) | REF |  |  |  |
| **3. Farm-level factors that may influence exposure** | | | | | | | | |
| **Experience in farming** | 5 to 10 years | 141 (22.4%) | 24 (15.7%) | 165 (21.1%) | 0.572 | 0.352 | 0.928 | 0.024 |
|  | 10 to 15 years | 72 (11.4%) | 3 (2.0%) | 75 (9.6%) | 0.140 | 0.043 | 0.453 | 0.001 |
|  | 15 to 20 years | 60 (9.5%) | 20 (13.1%) | 80 (10.2%) | 1.119 | 0.646 | 1.941 | 0.688 |
|  | More than 20 years | 356 (56.6%) | 106 (69.3%) | 462 (59.1%) | REF |  |  |  |
| **Distance to neighbouring farm** | Less than 1 km | 310 (51.7%) | 103 (65.6%) | 413 (54.6%) | 536.833 | 0.000 |  | 0.998 |
|  | 1-5 km | 224 (37.3%) | 50 (31.8%) | 274 (36.2%) | 360.705 | 0.000 |  | 0.998 |
|  | 6-10 km | 26 (4.3%) | 4 (2.5%) | 30 (4.0%) | 248.667 | 0.000 |  | 0.998 |
|  | Over 10 km | 30 (5.0%) | 0 (0.0%) | 30 (4.0%) | REF |  |  |  |
|  | Do not know | 10 (1.7%) | 0 (0.0%) | 10 (1.3%) | 1.000 | 0.000 |  | 1.000 |
| **Wildlife interaction within 2yrs** | No | 320 (55.4%) | 54 (38.8%) | 374 (52.2%) | REF |  |  |  |
|  | Yes | 258 (44.6%) | 85 (61.2%) | 343 (47.8%) | 1.952 | 1.337 | 2.850 | 0.001 |
| **Farm located near international border.** | No | 410 (65.2%) | 113 (73.9% | 523 (66.9%) | REF |  |  |  |
|  | Yes | 219 (34.8%) | 40 (26.1%) | 259 (33.1%) | 0.663 | 0.446 | 0.985 | 0.042 |
| **Farm located near National Park** | No | 352 (56.5%) | 61 (38.4%) | 413 (52.8%) | REF |  |  |  |
|  | Yes | 271 (43.5%) | 98 (61.6%) | 369 (47.2%) | 2.087 | 1.461 | 2.981 | <0.001 |
| **Distance to the nearest National Park** | Less than 5 km | 156 (58.6%) | 44 (47.3%) | 200 (55.7%) | 0.395 | 0.214 | 0.729 | 0.003 |
|  | 5-10 km | 49 (18.4%) | 20 (21.5%) | 69 (19.2%) | 0.571 | 0.275 | 1.187 | 0.133 |
|  | More than 10 km | 35 (13.2%) | 25 (26.9%) | 60 (16.7%) | REF |  |  |  |
|  | Do not know | 26 (9.8%) | 4 (4.3%) | 30 (8.4%) | 0.215 | 0.067 | 0.695 | 0.010 |
| **4. Farm management practices and association with FMD seropositivity** | | | | | | | | |
| **Herd size of cattle kept** | Large herd (over 50) | 295 (61.1%) | 84 (70.0%) | 379 (62.9%) | 1.032 | 0.564 | 1.889 | 0.918 |
|  | Medium size (21-50) | 130 (26.9%) | 20 (16.7%) | 150 (24.9%) | 0.558 | 0.270 | 1.153 | 0.115 |
|  | Small herd (1-20) | 58 (12.0%) | 16 (13.3%) | 74 (12.3%) | REF |  |  |  |
| **Goats mixed up with sheep** | Yes | 312 (48.3%) | 67 (41.6%) | 379 (47.0%) | 0.763 | 0.538 | 1.082 | 0.129 |
|  | No | 334 (51.7%) | 94 (58.4%) | 428 (53.0%) | REF |  |  |  |
| **Number of sheep kept** | Large herd (over 30) | 43 (13.8%) | 27 (40.3%) | 70 (18.5%) | 3.898 | 2.043 | 7.438 | 0.000 |
|  | Medium herd (16-30) | 120 (38.5%) | 16 (23.9%) | 136 (35.9%) | 0.828 | 0.421 | 1.629 | 0.584 |
|  | Small herd (1-15) | 149 (47.8%) | 24 (35.8%) | 173 (45.6%) | REF |  |  |  |
| **Number of goats kept** | Large herd (over 30) | 479 (74.1%) | 139 (86.3%) | 618 (76.6%) | 1.161 | 0.382 | 3.529 | 0.793 |
|  | Medium herd (16-30) | 151 (23.4%) | 18 (11.2%) | 169 (20.9%) | 0.477 | 0.144 | 1.583 | 0.226 |
|  | Small herd (1-15) | 16 (2.5%) | 4 (2.5%) | 20 (2.5%) | REF |  |  |  |
| **Sheep + goats herd size** | Large herd (over 30) | 531 (82.2%) | 146 (90.7%) | 677 (83.9%) | 1.100 | 0.362 | 3.340 | 0.867 |
|  | Medium herd (16-30) | 99 (15.3%) | 11 (6.8%) | 110 (13.6%) | 0.444 | 0.126 | 1.567 | 0.207 |
|  | Small herd (1-15) | 16 (2.5%) | 4 (2.5%) | 20 (2.5%) | REF |  |  |  |
| **Keep pigs on farm** | No | 605 (95.0%) | 148 (95.5%) | 753 (95.1%) | REF |  |  |  |
|  | Yes | 32 (5.0%) | 7 (4.5%) | 39 (4.9%) | 0.894 | 0.387 | 2.066 | 0.794 |
| **Bought in animals recently** | No | 252 (37.8%) | 82 (49.7%) | 334 (40.1%) | REF |  |  |  |
|  | Yes | 415 (62.2%) | 83 (50.3%) | 498 (59.9%) | 0.615 | 0.436 | 0.866 | 0.005 |
| **Number purchased** | Large Num bought (16-30) | 56 (13.5%) | 4 (4.8%) | 60 (12.0%) | 0.442 | 0.151 | 1.295 | 0.137 |
|  | Medium Num bought (6-15) | 136 (32.8%) | 43 (51.8%) | 179 (35.9%) | 1.959 | 1.198 | 3.202 | 0.007 |
|  | Small Num bought (1-5) | 223 (53.7%) | 36 (43.4%) | 259 (52.0%) | REF |  |  |  |
| **Make silage for cattle** | No | 463 (86.1%) | 133 (92.4%) | 596 (87.4%) | 1.959 | 1.011 | 3.795 | 0.046 |
|  | Yes | 75 (13.9%) | 11 (7.6%) | 86 (12.6%) | REF |  |  |  |
| **Make silage for shoats** | No | 540 (91.4%) | 146 (93.6%) | 686 (91.8%) | 1.379 | 0.683 | 2.782 | 0.370 |
|  | Yes | 51 (8.6%) | 10 (6.4%) | 61 (8.2%) | REF |  |  |  |
| **You move animals out of farm** | No | 341 (54.4%) | 92 (59.0%) | 433 (55.3%) | REF |  |  |  |
|  | Yes | 286 (45.6%) | 64 (41.0%) | 350 (44.7%) | 0.829 | 0.581 | 1.184 | 0.303 |
| **How frequent you move animals** | Daily | 160 (58.2%) | 30 (54.5%) | 190 (57.6%) | 1.687 | 0.372 | 7.654 | 0.498 |
|  | Monthly | 2 (0.7%) | 8 (14.5%) | 10 (3.0%) | 36.000 | 4.280 | 302.804 | 0.001 |
|  | Seasonally (esp. dry season) | 95 (34.5%) | 15 (27.3%) | 110 (33.3%) | 1.421 | 0.299 | 6.756 | 0.659 |
|  | Others | 14 (5.1%) | 0 (0.0%) | 14 (4.2%) | REF |  |  |  |
| **Move animals across border** | No | 82 (59.0%) | 18 (58.1%) | 100 (58.8%) | REF |  |  |  |
|  | Yes | 57 (41.0%) | 13 (41.9%) | 70 (41.2%) | 1.039 | 0.472 | 2.288 | 0.924 |
| **Share grazing land** | No | 24 (40.7%) | 6 (54.5%) | 30 (42.9%) | REF |  |  |  |
|  | Yes | 35 (59.3%) | 5 (45.5%) | 40 (57.1%) | 0.571 | 0.156 | 2.087 | 0.397 |
| **Graze communally** | Daily | 19 (54.3%) | 1 (20.0%) | 20 (50.0%) | 0.211 | 0.021 | 2.079 | 0.182 |
|  | Seasonally | 16 (45.7%) | 4 (80.0%) | 20 (50.0%) | REF |  |  |  |
| **Share grazing area in dry season** | No | 311 (50.9%) | 78 (56.9%) | 389 (52.0%) | REF |  |  |  |
|  | Yes | 300 (49.1%) | 59 (43.1%) | 359 (48.0%) | 0.784 | 0.540 | 1.139 | 0.202 |
| **Distance moved in dry season** | Less than 5 km | 150 (51.7%) | 40 (67.8%) | 190 (54.4%) |  |  |  |  |
|  | 5-10 km | 117 (40.3%) | 13 (22.0%) | 130 (37.2%) | 0.417 | 0.213 | 0.815 | 0.011 |
|  | More than 10 km | 23 (7.9%) | 6 (10.2%) | 29 (8.3%) | 0.978 | 0.373 | 2.565 | 0.964 |
| **Farm is fenced** | No | 322 (48.3%) | 82 (49.7%) | 404 (48.6%) | 1.059 | 0.753 | 1.488 | 0.744 |
|  | Yes | 345 (51.7%) | 83 (50.3%) | 428 (51.4%) | REF |  |  |  |
| **Fence can prevent animal intrusion** | No | 349 (54.6%) | 84 (54.9%) | 433 (54.7%) | 1.012 | 0.710 | 1.442 | 0.949 |
|  | Yes | 290 (45.4%) | 69 (45.1%) | 359 (45.3%) | REF |  |  |  |
| **Farm has a gate** | No | 392 (59.8%) | 96 (61.1%) | 488 (60.1%) | 1.056 | 0.739 | 1.509 | 0.765 |
|  | Yes | 263 (40.2%) | 61 (38.9%) | 324 (39.9%) | REF |  |  |  |
| **Footbath available on farm** | No | 576 (87.7%) | 143 (86.7%) | 719 (87.5%) | 0.914 | 0.551 | 1.515 | 0.728 |
|  | Yes | 81 (12.3%) | 22 (13.3%) | 103 (12.5%) | REF |  |  |  |
| **Footbath is being used** | No | 571 (92.7%) | 142 (93.4%) | 713 (92.8%) | 1.119 | 0.551 | 2.275 | 0.756 |
|  | Yes | 44 (7.1%) | 10 (6.6%) | 54 (7.0%) | REF |  |  |  |
| **Last FMD vaccination on farm** | <6 months ago, | 378 (68.2%) | 121 (76.6%) | 499 (70.1%) | 0.747 | 0.333 | 1.674 | 0.479 |
|  | >6 months to 1 year | 89 (16.1%) | 16 (10.1%) | 105 (14.7%) | 0.419 | 0.163 | 1.079 | 0.072 |
|  | >1-2 years | 47 (8.5%) | 11 (7.0%) | 58 (8.1%) | 0.546 | 0.197 | 1.515 | 0.245 |
|  | I don’t remember | 19 (3.4%) | 1 (0.6%) | 20 (2.8%) | 0.123 | 0.014 | 1.062 | 0.057 |
|  | Never vaccinated | 21 (3.8%) | 9 (5.7%) | 30 (4.2%) | REF |  |  |  |
| **Received FMD training** | Yes | 430 (64.5%) | 122 (73.9%) | 552 (66.3%) | REF |  |  |  |
|  | No | 237 (35.5%) | 43 (26.1%) | 280 (33.7%) | 0.639 | 0.436 | 0.937 | 0.022 |

REF = Reference category.

**Supplementary Table 4:** Results of multicollinearity assessment.

| **Variable Pair** | **Cramer's V** | **Significance** | **Interpretation** |
| --- | --- | --- | --- |
| FMD hist * Last FMD case | 0.970 | 0.000 | Very strong association |
| Observed any clin signs * Neigh. had FMD | 0.672 | 0.000 | Strong association |
| District * Farm located near international border | 0.863 | 0.000 | Very strong association |
| Wildlife interact (2yrs) * Farm located near NP. | 0.223 | 0.000 | Weak association |
| Farm exp * Dist. to nearest park | 0.355 | 0.000 | Moderate association |
| Farm exp * FMD history | 0.271 | 0.000 | Moderate association |
| Farm located near border * Wildlife interact (2yrs) | 0.657 | 0.000 | Strong association |
| Breed * District | 0.487 | 0.000 | Moderate to strong association |
| District * FMD history | 0.504 | 0.000 | Strong association |

**Supplementary Table 5**: Results of multivariable logistic regression analyses - seven different models evaluated.

| **Model 1** | Exp(B) | 95% C.I. for EXP(B) | | P value | Included Cases (n/N) | Hosmer-Lemeshow (p) | Nagelkerke R² | AIC | Accuracy |
| --- | --- | --- | --- | --- | --- | --- | --- | --- | --- |
|  |  | Lower | Upper |  |  |  |  |  |  |
| Crossbred goats | 0.514 | 0.308 | 0.856 | 0.011 | 657/832 | 0.809 | 0.224 | 607.29 | 0.791 |
| Local breed goats (ref) |  |  |  |  |  |  |  |  |  |
| Kasese district | 0.009 | 0.002 | 0.050 | 0.000 |  |  |  |  |  |
| Kiruhura district | 0.052 | 0.011 | 0.248 | 0.000 |  |  |  |  |  |
| Nakasongola district | 0.093 | 0.027 | 0.323 | 0.000 |  |  |  |  |  |
| Rakai district | 0.389 | 0.143 | 1.056 | 0.064 |  |  |  |  |  |
| Kiboga district (REF) |  |  |  |  |  |  |  |  |  |
| Farm located near national park (Yes) | 14.371 | 3.804 | 54.286 | 0.000 |  |  |  |  |  |
| Farm located near national park (No) (REF) |  |  |  |  |  |  |  |  |  |
| FMD hist (<6 months) | 6.628 | 3.578 | 12.278 | 0.000 |  |  |  |  |  |
| FMD hist (6-12 months) | 1.543 | 0.480 | 4.961 | 0.467 |  |  |  |  |  |
| FMD hist (>1 year) | 2.189 | 1.218 | 3.933 | 0.009 |  |  |  |  |  |
| FMD hist (Never had FMD) (REF) |  |  |  |  |  |  |  |  |  |
| **Model 2** | Exp(B) | 95% C.I. for EXP(B) | | P value | Included Cases (n/N) | Hosmer-Lemeshow (p) | Nagelkerke R² | AIC | Accuracy |
|  |  | Lower | Upper |  |  |  |  |  |  |
| Crossbred goats | 0.512 | 0.302 | 0.868 | 0.013 | 647/832 | 0.622 | 0.22 | 611.62 | 0.788 |
| Local breed goats (ref) |  |  |  |  |  |  |  |  |  |
| Kasese district | 0.009 | 0.001 | 0.054 | 0.000 |  |  |  |  |  |
| Kiruhura district | 0.051 | 0.010 | 0.268 | 0.000 |  |  |  |  |  |
| Nakasongola district | 0.086 | 0.022 | 0.339 | 0.000 |  |  |  |  |  |
| Rakai district | 0.399 | 0.144 | 1.107 | 0.078 |  |  |  |  |  |
| Kiboga district (REF) |  |  |  |  |  |  |  |  |  |
| Farm located near national park (Yes) | 15.488 | 3.980 | 60.267 | 0.000 |  |  |  |  |  |
| Farm located near national park (No) (REF) |  |  |  |  |  |  |  |  |  |
| FMD hist (<6 months) | 6.079 | 2.959 | 12.490 | 0.000 |  |  |  |  |  |
| FMD hist (6-12 months) | 1.442 | 0.414 | 5.023 | 0.566 |  |  |  |  |  |
| FMD hist (>1 year) | 2.080 | 1.123 | 3.851 | 0.020 |  |  |  |  |  |
| FMD hist (Never had FMD) (REF) |  |  |  |  |  |  |  |  |  |
| Neighb had FMD=Yes | 1.259 | 0.483 | 3.282 | 0.637 |  |  |  |  |  |
| Neighb had FMD=No (REF) |  |  |  |  |  |  |  |  |  |
| Received FMD training =Yes | 0.831 | 0.471 | 1.468 | 0.524 |  |  |  |  |  |
| Received FMD training =No (REF) |  |  |  |  |  |  |  |  |  |
| Bought in animals past 12 months (Yes) | 1.072 | 0.575 | 1.999 | 0.826 |  |  |  |  |  |
| Bought in animals past 12 months (No) (REF) |  |  |  |  |  |  |  |  |  |
|  |  |  |  |  |  |  |  |  |  |
| **Model 3** | Exp(B) | 95% C.I. for EXP(B) | | P value | Included Cases (n/N) | Hosmer-Lemeshow (p) | Nagelkerke R² | AIC | Accuracy |
|  |  | Lower | Upper |  |  |  |  |  |  |
| Crossbred goats | 0.499 | 0.296 | 0.840 | 0.009 | 647/832 | 0.053 | 0.219 | 609.02 | 0.788 |
| Local breed goats (ref) |  |  |  |  |  |  |  |  |  |
| Kasese district | 0.011 | 0.002 | 0.059 | 0.000 |  |  |  |  |  |
| Kiruhura district | 0.058 | 0.012 | 0.287 | 0.000 |  |  |  |  |  |
| Nakasongola district | 0.101 | 0.028 | 0.361 | 0.000 |  |  |  |  |  |
| Rakai district | 0.409 | 0.148 | 1.130 | 0.085 |  |  |  |  |  |
| Kiboga district (REF) |  |  |  |  |  |  |  |  |  |
| Farm located near national park (Yes) | 14.335 | 3.777 | 54.414 | 0.000 |  |  |  |  |  |
| Farm located near national park (No) (REF) |  |  |  |  |  |  |  |  |  |
| FMD hist (<6 months) | 5.932 | 2.898 | 12.139 | 0.000 |  |  |  |  |  |
| FMD hist (6-12 months) | 1.501 | 0.437 | 5.160 | 0.519 |  |  |  |  |  |
| FMD hist (>1 year) | 2.114 | 1.143 | 3.910 | 0.017 |  |  |  |  |  |
| FMD hist (Never had FMD) (REF) |  |  |  |  |  |  |  |  |  |
| Neighb had FMD=Yes | 1.180 | 0.462 | 3.018 | 0.729 |  |  |  |  |  |
| Neighb had FMD=No (REF) |  |  |  |  |  |  |  |  |  |
| Bought in animals past 12 months (Yes) | 1.110 | 0.604 | 2.039 | 0.737 |  |  |  |  |  |
| Bought in animals past 12 months (No) (REF) |  |  |  |  |  |  |  |  |  |
| **Model 4** | Exp(B) | 95% C.I. for EXP(B) | | P value | Included Cases (n/N) | Hosmer-Lemeshow (p) | Nagelkerke R² | AIC | Accuracy |
|  |  | Lower | Upper |  |  |  |  |  |  |
| Crossbred goats | 0.518 | 0.309 | 0.869 | 0.013 | 647/832 | 0.335 | 0.22 | 609.66 | 0.788 |
| Local breed goats (ref) |  |  |  |  |  |  |  |  |  |
| Kasese district | 0.009 | 0.001 | 0.053 | 0.000 |  |  |  |  |  |
| Kiruhura district | 0.049 | 0.010 | 0.241 | 0.000 |  |  |  |  |  |
| Nakasongola district | 0.084 | 0.022 | 0.327 | 0.000 |  |  |  |  |  |
| Rakai district | 0.393 | 0.143 | 1.083 | 0.071 |  |  |  |  |  |
| Kiboga district (REF) |  |  |  |  |  |  |  |  |  |
| Farm located near national park (Yes) | 15.369 | 3.954 | 59.738 | 0.000 |  |  |  |  |  |
| Farm located near national park (No) (REF) |  |  |  |  |  |  |  |  |  |
| FMD hist (<6 months) | 6.146 | 3.008 | 12.559 | 0.000 |  |  |  |  |  |
| FMD hist (6-12 months) | 1.391 | 0.415 | 4.661 | 0.593 |  |  |  |  |  |
| FMD hist (>1 year) | 2.060 | 1.119 | 3.793 | 0.020 |  |  |  |  |  |
| FMD hist (Never had FMD) (REF) |  |  |  |  |  |  |  |  |  |
| Neighb had FMD=Yes | 1.254 | 0.482 | 3.266 | 0.642 |  |  |  |  |  |
| Neighb had FMD=No (REF) |  |  |  |  |  |  |  |  |  |
| Received FMD training =Yes | 0.822 | 0.470 | 1.439 | 0.493 |  |  |  |  |  |
| Received FMD training =No (REF) |  |  |  |  |  |  |  |  |  |
| **Model 5** | Exp(B) | 95% C.I. for EXP(B) | | P value | Included Cases (n/N) | Hosmer-Lemeshow (p) | Nagelkerke R² | AIC | Accuracy |
|  |  | Lower | Upper |  |  |  |  |  |  |
| Crossbred goats | 0.506 | 0.303 | 0.846 | 0.009 | 647/832 | 0.705 | 0.219 | 608.13 | 0.788 |
| Local breed goats (ref) |  |  |  |  |  |  |  |  |  |
| Kasese district | 0.011 | 0.002 | 0.059 | 0.000 |  |  |  |  |  |
| Kiruhura district | 0.054 | 0.011 | 0.259 | 0.000 |  |  |  |  |  |
| Nakasongola district | 0.099 | 0.028 | 0.353 | 0.000 |  |  |  |  |  |
| Rakai district | 0.401 | 0.146 | 1.101 | 0.076 |  |  |  |  |  |
| Kiboga district (REF) |  |  |  |  |  |  |  |  |  |
| Farm located near national park (Yes) | 14.036 | 3.721 | 52.945 | 0.000 |  |  |  |  |  |
| Farm located near national park (No) (REF) |  |  |  |  |  |  |  |  |  |
| FMD hist (<6 months) | 6.015 | 2.948 | 12.274 | 0.000 |  |  |  |  |  |
| FMD hist (6-12 months) | 1.428 | 0.429 | 4.752 | 0.561 |  |  |  |  |  |
| FMD hist (>1 year) | 2.085 | 1.132 | 3.840 | 0.018 |  |  |  |  |  |
| FMD hist (Never had FMD) (REF) |  |  |  |  |  |  |  |  |  |
| Neighbour had FMD=Yes | 1.168 | 0.458 | 2.984 | 0.745 |  |  |  |  |  |
| Neighbour had FMD=No (REF) |  |  |  |  |  |  |  |  |  |

(REF) – Reference category for the variable.
