# Supplementary material for: Epidemiology of Foot‐and‐Mouth Disease in Goats in Uganda: A Risk‐Based Approach
Source: Transbound Emerg Dis. 2026 Apr 15;2026:2808139. doi: 10.1155/tbed/2808139 (PMC13080341; doi:10.1155/tbed/2808139)
Supplement: Supplementary file 3 — Supporting Information 3 File S2: Sample collection form used to identify the sample and capture animal biodata and geographical information. [file TBED-2026-2808139-s003.docx]

**Supplementary File 2**

**Sample Collection Form**
*Foot-and-Mouth Disease Study – Uganda*

1. **General Information:** District: ……………………… Subcounty: …………………….. Parish: …………………… Village: …………………………………………………………
2. **Farm Information: Farm Owner**: ………………………….. **Unique ID**: …………………………………. **Sampling Date**: …………………………. **Farm ID**: ………………
3. **Animal Information**

| **Animal No.** | **Animal ID** | **Sample ID** | **Species (cattle/goat/sheep)** | **Breed (local/cross/exotic)** | **Age** | **Sex** | **BCS** | **Comments** |
| --- | --- | --- | --- | --- | --- | --- | --- | --- |
|  |  |  |  |  |  |  |  |  |
|  |  |  |  |  |  |  |  |  |
|  |  |  |  |  |  |  |  |  |
|  |  |  |  |  |  |  |  |  |
|  |  |  |  |  |  |  |  |  |
|  |  |  |  |  |  |  |  |  |
|  |  |  |  |  |  |  |  |  |
|  |  |  |  |  |  |  |  |  |
|  |  |  |  |  |  |  |  |  |
|  |  |  |  |  |  |  |  |  |
|  |  |  |  |  |  |  |  |  |
|  |  |  |  |  |  |  |  |  |
|  |  |  |  |  |  |  |  |  |
|  |  |  |  |  |  |  |  |  |
|  |  |  |  |  |  |  |  |  |
|  |  |  |  |  |  |  |  |  |
